# Automated BIM-based structural design and cost optimization model for reinforced concrete buildings

**DOI:** 10.1038/s41598-022-26146-6

**Published:** 2022-12-14

**Authors:** Mohamed Sherif, Khaled Nassar, Ossama Hosny, Sherif Safar, Ibrahim Abotaleb

**Affiliations:** Department of Construction Engineering, School of Sciences and Engineering, The American University in Cairo, New Cairo, Egypt

**Keywords:** Civil engineering, Computational science, Scientific data

## Abstract

The process of optimizing building designs requires developing several architectural and structural layout alternatives. Traditionally, limited number of design iterations can be conducted manually, which is time consuming and results in non-optimum designs in terms of limited functionality or high costs. The goal of this research is to develop an advanced Building Information Modeling (BIM) model for automating and optimizing design of building layouts and structural elements to reach minimum construction cost while abiding by the functionality constraints of the architectural design. The developed model integrates concepts from structural design, BIM modeling, and computer programming into one advanced optimization framework. The model was tested and validated in 11 case studies and is found to reduce the structural materials cost by up to 15% per floor without compromising the defined space requirements.

## Introduction

The design process for buildings can include several stages, such as conceptual design, schematic design, and detailed design. It is characterized as multi-disciplinary, multi-objective, and multi-parameter, where it necessitates the management of a wide range of data and parameters across disciplines over time. The rise of information and communication technology (ICT) has elevated the capabilities of engineers to manage information and enable seamless handling of complexities in the design process^1^. The concept of working with digital representations of a building, represented in Building Information Modelling (BIM), has been recognized as the cornerstone for information management throughout ICT advancements in the construction sector^2^. Working with a digital representation of a building's physical and functional features attempts to make design knowledge transferable between different software components and diverse teams^3^. BIM systems are based on the parametric definition of objects, where they can differentiate between different elements of a structure (beams, columns, slabs, windows, doors, and walls) by analyzing their attributes such as usage, structures, and functions and studying their parametric characteristics. Relations and connections between the attributes of the structural elements are then developed to detect any alterations and discoordination in models^3^. BIM systems range from simple 2D models to nowadays 9D models; where they can include integrative data such as dimensions, spaces, volumes, materials, time schedules, cost estimates, energy efficiency, facility management, safety aspect, and lean aspects^4^. BIM presents a growing technological and procedural shift in construction operations^5^. Enhanced identification and omissions of errors, early collaboration of several concurrent disciplines, and improved building quality and performance are key benefits that result from BIM-based workflows^6^.

While a BIM-based workflow should enable overall information management throughout the lifecycle of a building, it is still significantly reliant on manual intervention^7^. As a result, there has been a growth in interest in automated workflows based on BIM to promote its usage and improve its potential efficiency^8^. For example^9^, developed a data model for integrating risk assessment of building conditions into BIM; where they automated the data transfer process and improve consistency and dependability for better visualization of conditions and causality analysis. El Mourabit^10^ developed a software for automating and optimizing concrete beam bridges. In another attempt^11^, investigated the automation trends of bridge design and highlighted the importance of automation in structural design in general as an important area to tackle. Earlier^12^, presented an optimization model for optimizing the design of prestressed concrete bridges. Other optimization models for design of bridges include those of^13–16^.

When it comes to buildings, design optimization models are not abundant and there is a need for further research in this area. For example^17^, developed a model for optimizing the cost of precast concrete slabs using evolutionary algorithms. Their model uses inputs such as the floor dimensions and live load to eventually provide structural design alternatives and arrange them. This model is able to design parameters such as the layout, dimensions, and reinforcement of the precast slabs. In another research^18^, developed a model for automating the design of flat slabs in concrete buildings using a hybrid optimization method. Other similar models are those of^19–22^.

There is a gap when it comes to BIM optimization models for traditional concrete systems such as solid slabs and flat slabs, which can be attributed to the complexities involved with their designs when compared to linear and straight-forward elements such as bridge beams. In addition, all of the discussed previous research concerning building design assumes fixed dimensions or fixed layouts of the floor slabs. In reality, structural engineers should have some flexibility to discuss the building layouts and may propose changes to the architects in order to optimize the structural design. In other words, there is some flexibility in the architectural design to shift some walls and columns within boundaries specified by the architect to fulfill certain functionality constraints to achieve optimum design. As such, optimization models should not assume fixed room dimensions. This research attempts to cover the above-mentioned gaps.

## Research goal

The goal of this research is to develop a model for automating and optimizing design of building layouts and structural elements for reaching minimum construction cost while abiding by the functionality constraints of the architectural design. The outputs from this model can have multiple uses, including developing an automated optimization framework for integrating the architectural and structural design of buildings, reaching optimum utilization of the functionality of the architectural design, and interpreting the optimum cost savings of the structural elements during the conceptual design phase of the construction projects. This framework can support the decision-making process between the architectural and structural design aspects in determining the best design alternative that is safe, satisfies architectural design and minimize cost. The framework maps the design principles and procedures from the international standard building codes into a developed mathematical model that can design different structural elements, including indeterminate structural elements. It also can act as a preliminary cost estimating tool that can evaluate the various design alternatives compared to a set budget.

## Methodology

**Figure 1:**
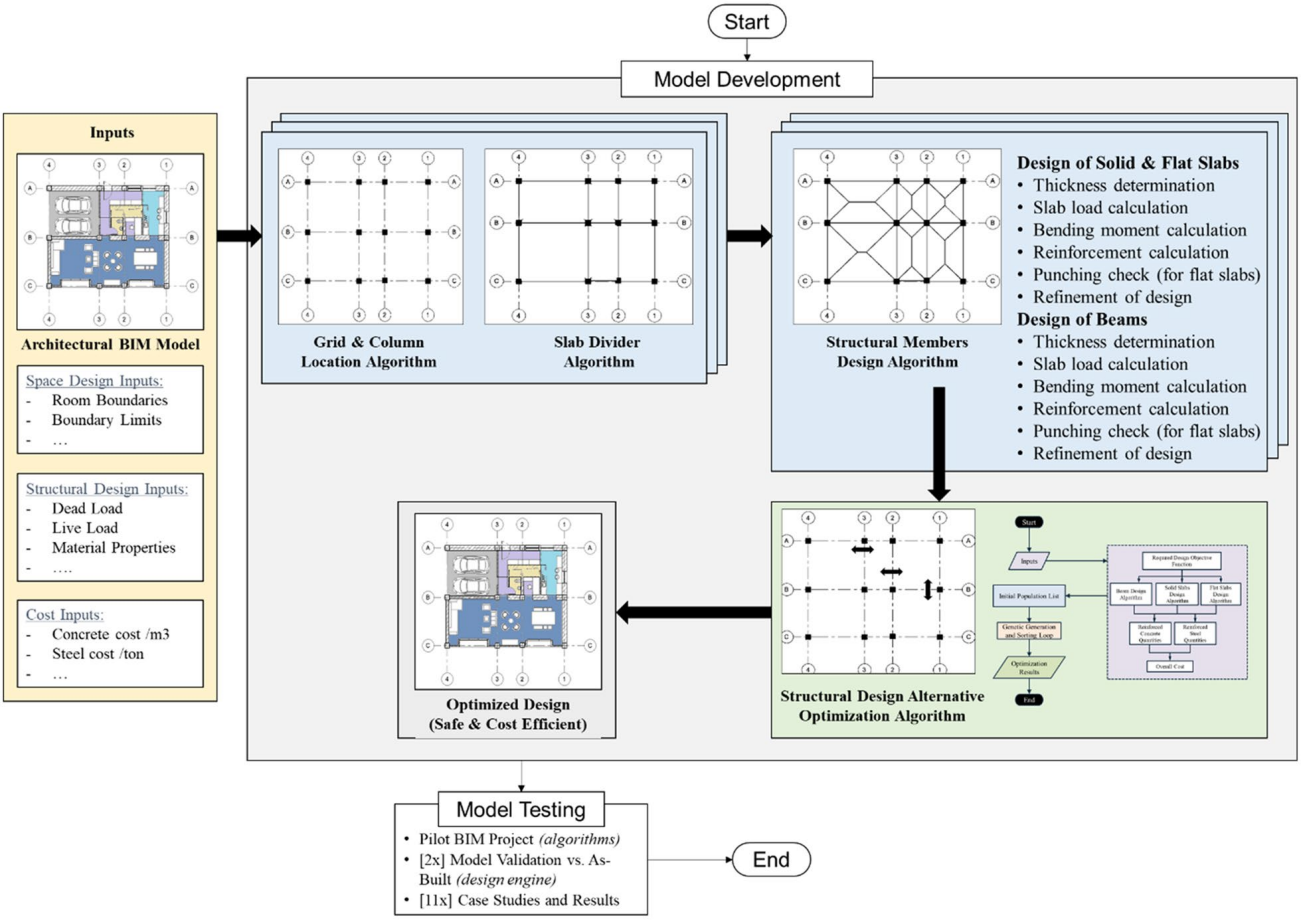
Research methodology.

The research utilizes and integrates concepts from structural design, BIM modeling, and computer programming into one advanced optimization framework. The research methodology is demonstrated in Fig. 1. First, a Cartesian coordinate system workflow is developed. In this workflow, algorithms are developed and used to convert the BIM architectural design into a more advanced state which model automated structural design. The inputs to this module are the architectural design, the architectural space limits such as different rooms boundaries and boundaries limits in both 2D directions, and the structural design inputs, which are mainly the required loads Material properties. The developed algorithms in this module are: grid and structural columns detection, Cartesian point arrangement, determination of geometric levels, and division of structural slabs. Details of these steps are described later in the manuscript. Second, a module for automating design of concrete it developed. In such module, automated design of slabs (solid and flat slabs) and beams is performed according to codes of practice. Third, an optimization module is developed where the user can choose their required design objective function such as optimizing the concrete quantities, steel quantities, or both. The model chooses a random initial population, then genetic generation and sorting loops to reach the required optimization results. The main idea is to allow the software to examine the proposed system and layout and perform optimization that includes repeated structure design. Each time a structural design is reached, a new model cost is computed then iterations take place to reach the final optimum design that is safe, applicable, and cost-efficient. After that, the developed framework is tested on different case studies to validate its effectiveness in reflecting the desired results. Detailed description is provided later in the manuscript.

The scope of work in this research is limited to:

- Structural design of projects that are natively designed with reinforced concrete elements in BIM. Other types of structural elements such as structural steel and timber are excluded.
- The structural design algorithms for slab elements can handle rectilinear architectural slabs only. Other irregular shaped slabs are excluded.
- The structural design algorithms can design and alternate between solid slab and flat slab structural systems as well as reinforced beams structural systems following the design principles and procedures according to the Egyptian Code of Practice (ECP) because the research was conducted in its premises. However, the Equations used in the structural design can be easily changed to fit any other codes such as the ACI 318 (for the US), or the British Standard BS 8110 (for the UK).

**Figure 2:**
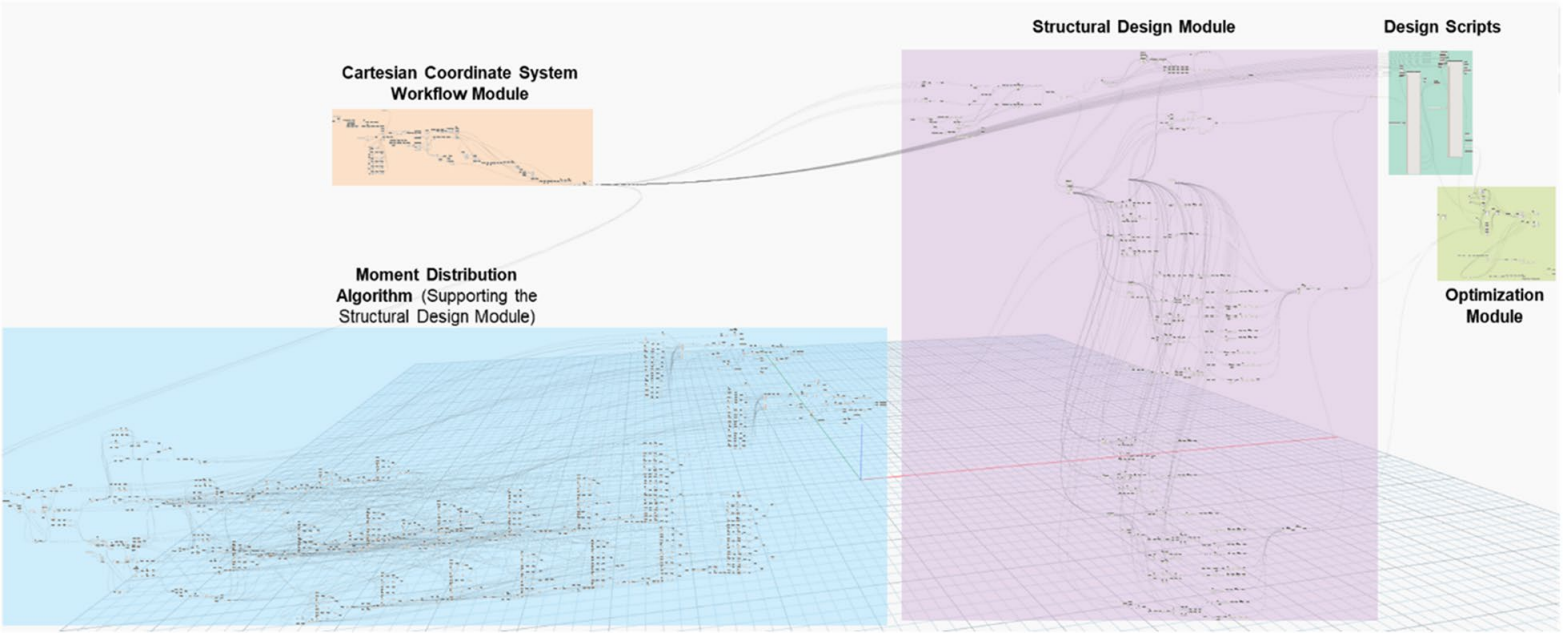
Overall model screenshot from the dynamo window.

The algorithms were coded in a BIM environment using design script visual programming language, where Autodesk Revit was used as the BIM environment and Dynamo add-in as design script compiler in Autodesk Revit. Dynamo and Python were used in this research due to its strong retrieving relations and manipulation capabilities with the Revit databases, where all model related data can be used in the interest of the optimization. Figure 2 shows a screenshot of the overall model on Dynamo; where the environment consists of node that process inputs into outputs. As the developed model is complex and large, one Figure cannot show it and the full details and Equations of each node cannot be described in the manuscript due to space limitations. However, in the following sections, the authors describe the key algorithms and processes that enable readers to understand the work and replicate it with a good level of aggregation. The full model is available to interested researchers by request.

## Cartesian coordinate system workflow module

### Grid and structural columns detection

**Figure 3:**
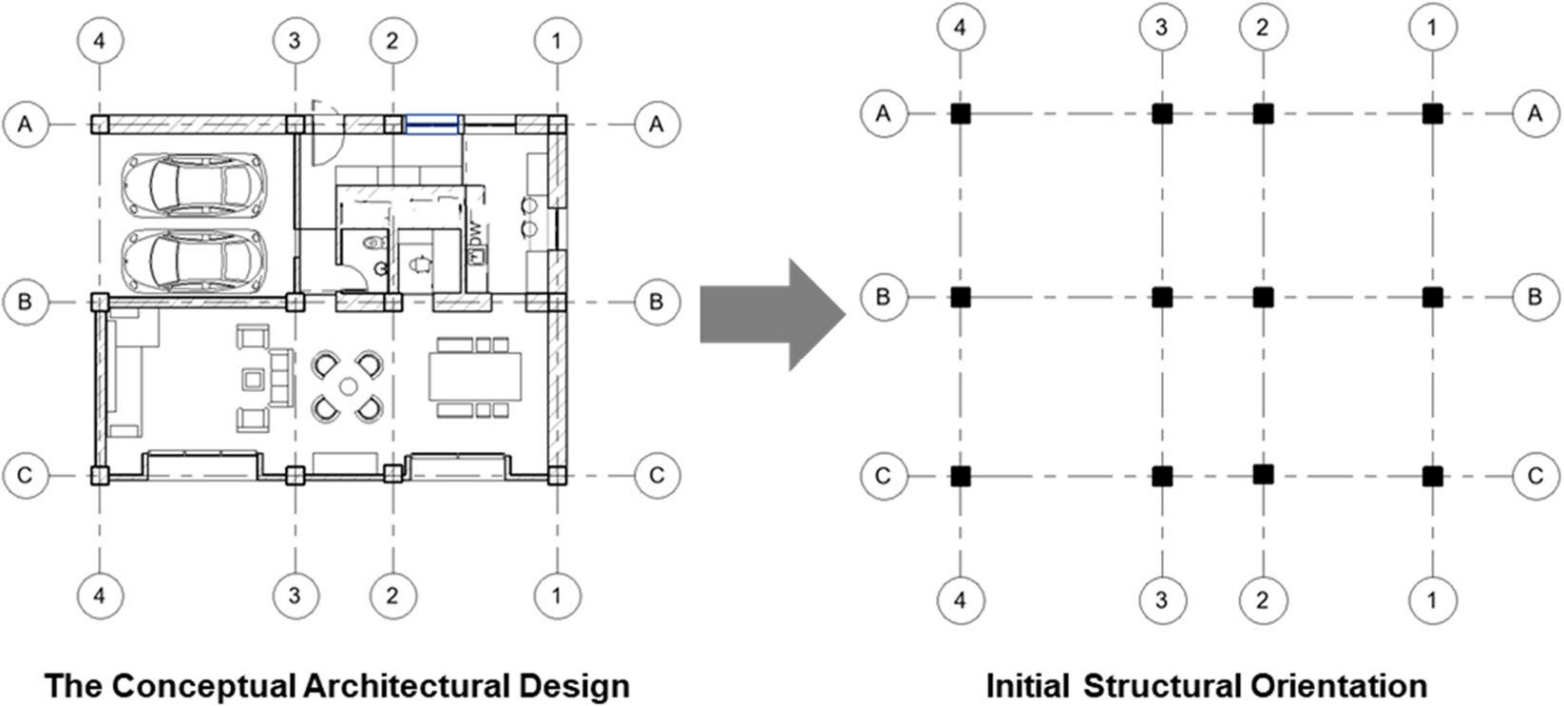
Grid and structural columns detection.

The architect first provides the conceptual architectural design for the floor plan; then, the model retrieves the initial location of the structural columns and the grid intersections as the primary inputs (Fig. 3), these data represent the main layout of the structural system. The initial architectural grid prepared by the user is used as a model input to prepare the structural grid which has the columns’ location marks, and the location of other structural elements, in other words, the model uses the architectural grid as one of the inputs to develop the structure grid for the analysis. The BIM environment makes the model generic to be applied to different building types with different functions and layouts. Also, the proposed model can be implemented on consequence levels of the building with the change in the relative loads and the implementation of different design parameters from one level to the next. For example, the loads applied on the columns of the ground floor of the building are more than the loads applied on the roof floor or, as stated by the structural engineer. The user can define these changes from one level to the next with the objective of optimized cost per level.

### Cartesian point arrangement

The followed Cartesian system requires specific vectoral arrangement for every element in the system such as grids, column’s locations and intersection points. This arrangement is then represented in a matrix form. After arranging the grid lines in both global directions and detecting the dimension variables, the model evaluates the intersection points between the grid lines in both directions and presents them as illustrated in Eq. (1), where each row contains the points on the same grid line. These intersection points are then evaluated to determine if each of these points has a column location mark or not. The intersection points with the column location marks are the necessary points for the model because the structural columns are the essential elements for supporting all the proposed systems. This matrix has three levels of information; level 1 presents the points of intersection, level 2 presents all the grid lines in X and Y directions, and level 3 presents the random populations necessary for the optimization process. Each population consists of certain random dimensions for the X and Y grid lines within the specified range. The model evaluates the intersection points of each population and forms the leveled matrix list.

1 $$IP = { }\left[ {\begin{array}{*{20}c} {I_{11} } & {I_{12} } & \cdots & {I_{1m} } \\ {I_{21} } & {I_{22} } & \cdots & {I_{1m} } \\ \vdots & \vdots & \ddots & \vdots \\ {I_{n1} } & {I_{n2} } & \cdots & {I_{nm} } \\ \end{array} } \right]$$

where n is the number of Gird Lines and m is the number of Intersection points per line.

### Geometric levels determination

The model is developed to work on different levels of the structure; the need for the structural columns decreases as the model goes upward, and there is a structural concept of reducing the dimensions of columns as the load decrease upward. To implement this concept, the model uses a geometry translate approach (Node) where the intersection points in the base level are translated to the next levels of the model (Ground level, first level, etc.). The model evaluates the intersection of these points with the columns again. If the intersection exists, the intersection point is retaken into consideration in the next level. If the intersection is not valid, the intersection point is removed from the matrix. The final list is represented in Eq. (2).

2 $$LV = { }\left[ {\begin{array}{*{20}c} {L_{11} } & {L_{12} } & \cdots & {L_{1m} } \\ {L_{21} } & {L_{22} } & \cdots & {L_{2m} } \\ \vdots & \vdots & \ddots & \vdots \\ {L_{n1} } & {L_{n2} } & \cdots & {L_{nm} } \\ \end{array} } \right]$$

where n is the number of model levels and m is the number of Intersection points per level.

### Division of structural slab

**Figure 4:**
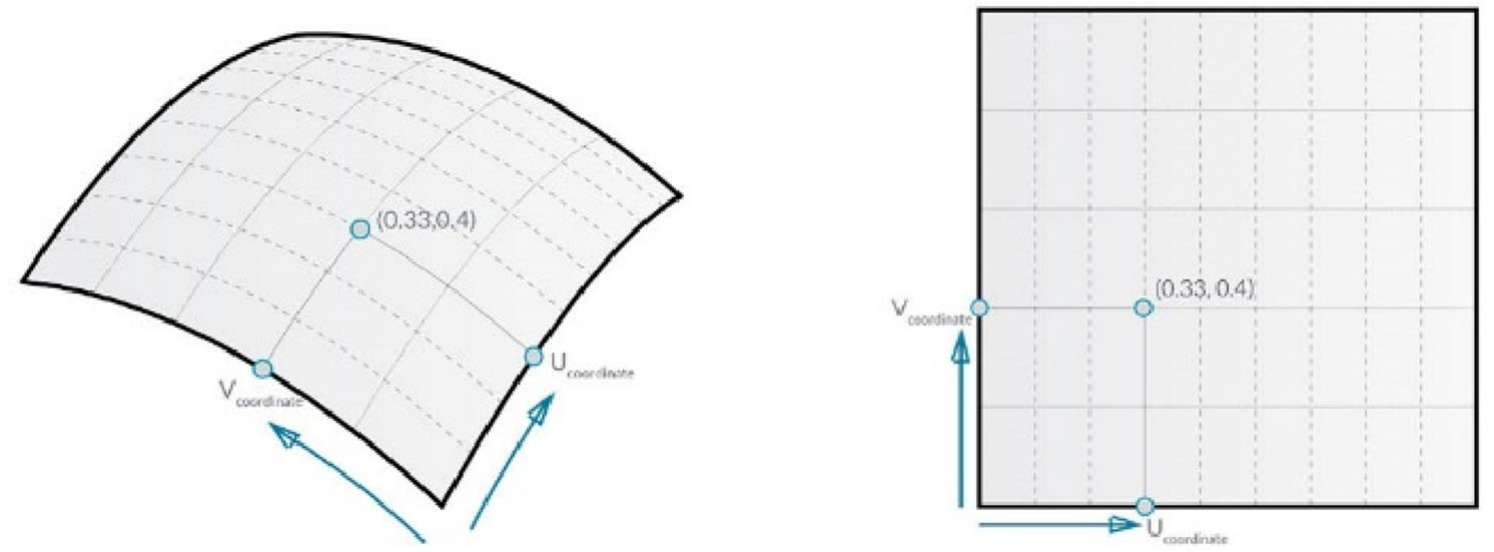
Surface-point as parameter subdivision.

As mentioned before, the user determines the required level to work on, starting from the bottom level till the top level. This extra feature enables the user to deal with a better realistic representation of construction projects where the reduction in the structural columns is implemented in a scientific method. Also, the matrices of the Eqs. 3, 4, 5 and 6 below are applied on different levels: level 01 represents the intersection points or columns’ locations, level 02 represents the model's levels or floors’ levels, and level 03 represents the optimization population or global coordinates. The structural elements design takes place on the slabs confined by each 4-columnson the structure plan. That is why the overall structural slab needs to be divided to undergo load division, analysis, and design. The method followed for such division is the “Surface-Point at parameter” technique^23^; where the surfaces is subdivided relative to a parametric Cartesian location of points on the surface, as shown in Fig. 4. Each point is defined by local Cartesian coordinates (U, V), which are values between 0 and 1 related to the surface's overall length and width. The process’s details are explained as follows.

The model detects and divides the overall surface dimensions based on the intersection points, then the X and Y dimensions are arranged in two cumulative dimensions vectors. The last element of the vector is the overall distance in each direction. A remapping function is then applied on each vector; the cumulative distances in each direction are remapped between zero and one. These two vectors which are shown in Eqs. 3 and 4 represent the location surface dimensions (the U and V parameters) as an output for the surface-points division. This process will be defined as “Surface-Points at parameter” and will be used as an independent function in the following steps. This function is used for local division of the structural slabs and detection of columns’ locations.

3 $$Local{ }U = { }\left[ {\begin{array}{*{20}c} 0 \\ {U_{21} } \\ \vdots \\ {U_{m1} } \\ 1 \\ \end{array} } \right]$$

4 $$Local{ }V = { }\left[ {\begin{array}{*{20}c} 0 \\ {V_{21} } \\ \vdots \\ {V_{n1} } \\ 1 \\ \end{array} } \right]$$

where m is the number of cumulative variables in the U local direction of each subdivided slab and n is the number of cumulative variables in the V location direction, all variables are between 0 and 1.

The model patches a surface between the four sides of the outer global dimensions and uses the U and V vectors to determine the inner columns' location points represented by the intersection points. Then the model uses the function “Surface-Points at parameter” to divide the overall surface by the (U, V) Cartesian coordinate system. The final matrix is presented in Eq. (5), where each row consists of points representing the columns arranged on the same line.

5 $$SP = { }\left[ {\begin{array}{*{20}c} {SP_{11} } & {SP_{12} } & \cdots & {SP_{1m} } \\ {SP_{21} } & {SP_{22} } & \cdots & {SP_{2m} } \\ \vdots & \vdots & \ddots & \vdots \\ {SP_{n1} } & {SP_{n2} } & \cdots & {SP_{nm} } \\ \end{array} } \right]$$

where n is the number of column lines in the Grid System and m is the number of columns location points per line.

**Figure 5:**
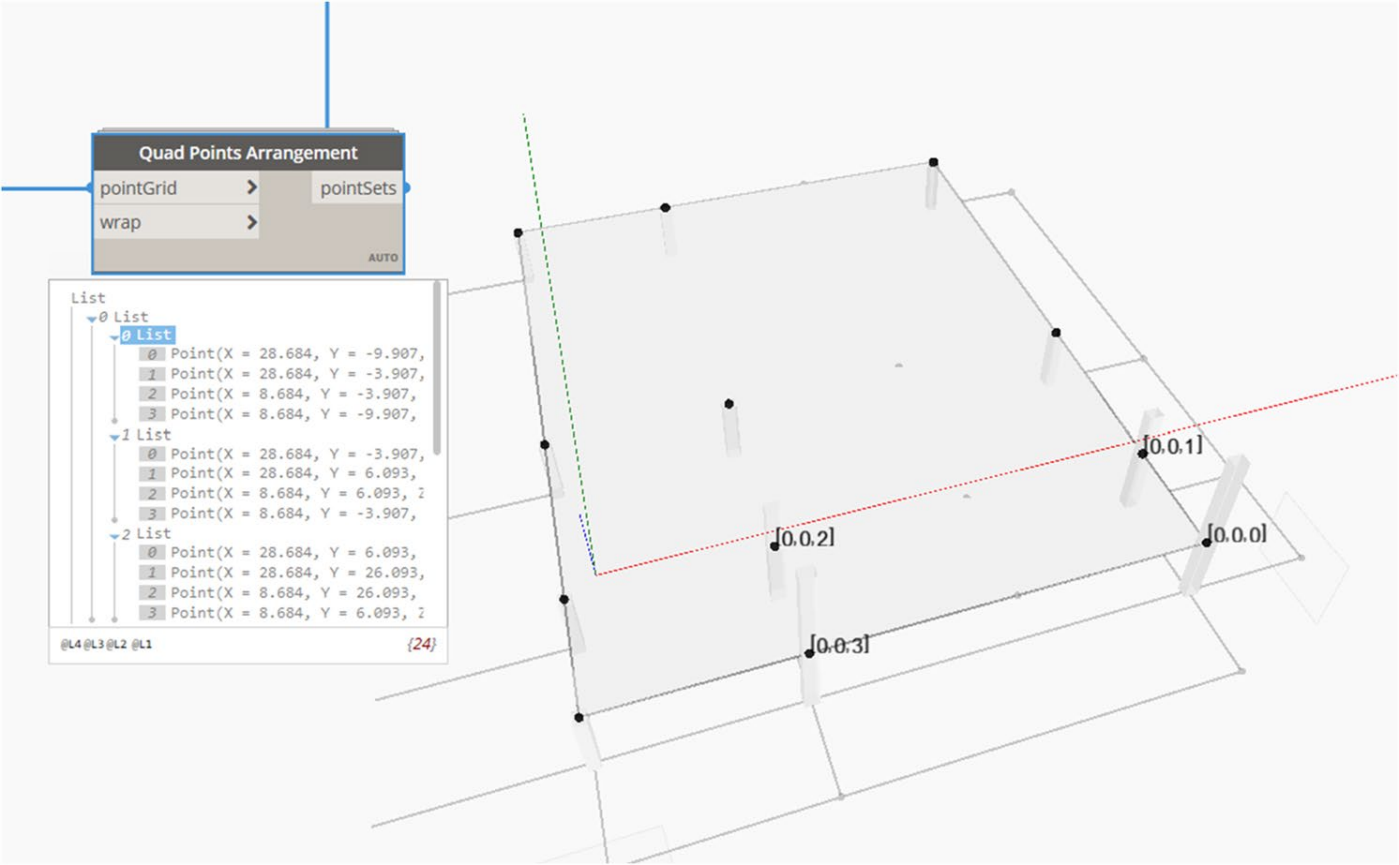
Structural slab quadratic arrangement.

This matrix, as previously illustrated, works on levels where the highest level represents the optimization population or global coordinates (variables). The model iterates the “Surface-Points at parameter” technique consecutive times to precisely ensure that the grid intersection points match the columns' location marks and that the grid system is inside the boundary limits of the analyzed BIM model. After dividing the overall slab into parametric points representing the columns, these points are arranged into a quadratic form. Each group of 4 points represents a structural subdivided slab carried by the four columns and undergoes load division and design. The model performs mathematical operations to reach the final quadratic arrangement of the slab division points, as shown in Eq. (6). Each row represents a subdivided slab and contains four quadratic points representing the slab's columns, as shown in Fig. 5.

6 $$Quad\,Points = { }\left[ {\begin{array}{*{20}c} {P_{11} } & {P_{12} } & \cdots & {P_{14} } \\ {P_{21} } & {P_{22} } & \cdots & {P_{24} } \\ \vdots & \vdots & \ddots & \vdots \\ {P_{n1} } & {P_{n2} } & \cdots & {P_{n4} } \\ \end{array} } \right]$$

where n is the number of subdivided quadratic slabs in the proposed plan.

**Figure 6:**
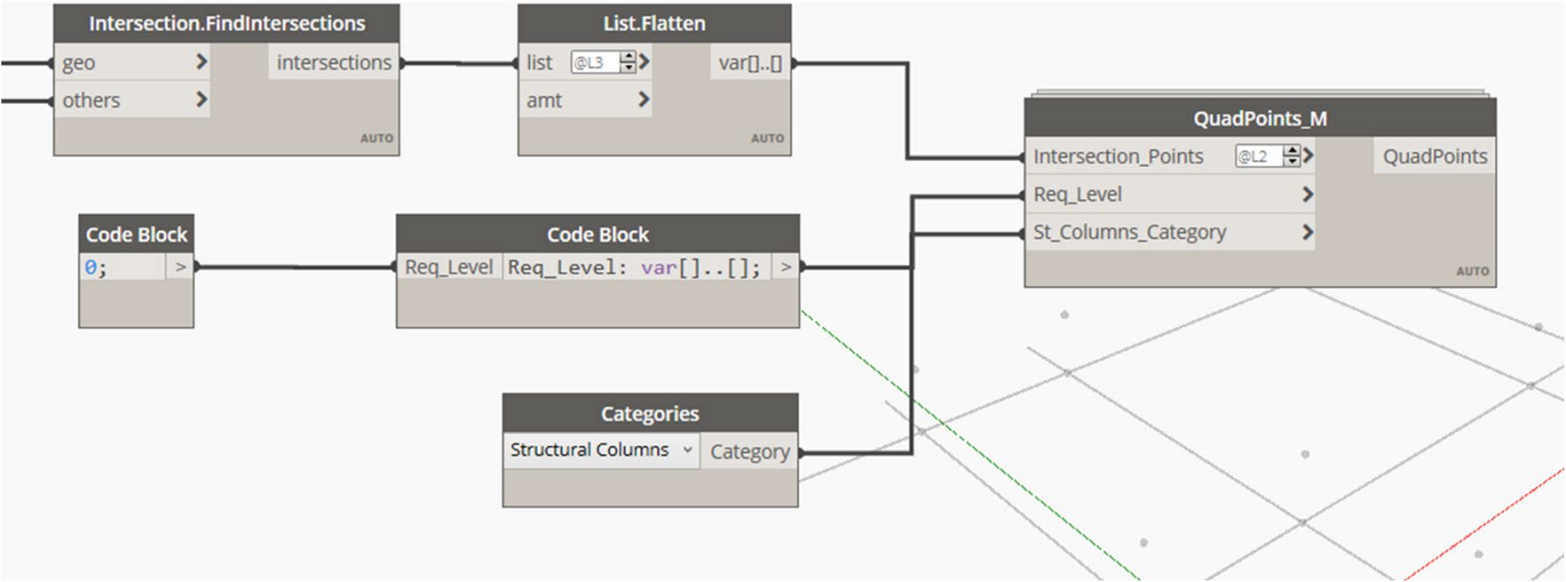
Subdivided quad points arrangement custom node (Screenshot from the coding on Dynamo).

All the previous steps and Equation nodes are grouped into a custom node to be used with the different populations in the optimization model and on the different levels. The inputs for this custom node will be the intersection points of the grid system representing the columns' location marks, the required level of floor defined by the user, and the structural columns category, which is the type of columns used in the model (Structural Columns) as presented in Fig. 6.

After Cartesian arrangement, each of the 4-Quadratic slab points group is used to create a group of polygon matrices to calculate the area within each polygon and be used in the structural slab design as will be described in the following sections.

## Structural design module

### Automated design of solid and flat slabs

The development of the design node of the solid slab or the flat slab starts by defining the required structural inputs by the engineer such as the live load, flooring load, ultimate stress of concrete and yielding stress of steel. These variables are collected in one custom node, which the user can change. This input variable custom node with the polygons' dimension interpreted by the model is the elements necessary to start the design as will be described afterward. The model flexibility to change the variables of the mentioned structural constants make it more generic and applicable to different architectural plans with minimum restrictions. The live and flooring loads can be defined as a sequence or range nodes, where the user can select the required values before running the optimization model. The model retrieves the X and Y dimension of each subdivided slab and uses this data to calculate the r-factor, the ratio between the long dimension and the short dimension of the slab. Based on this ratio the structural thickness of each slab is calculated according to the applied code of practice. After computing the distributed loads on slabs, the model calculates the structural bending moment based on the Equations provided in the code of practice. This is the simplified method, which relies on parametric Equations for calculation of loads and moments rather than conducting detailed structural analysis using finite element. All equations used in the model are extracted from the Egyptian Code of Practice (ECP) for Design and Construction of Reinforced Concrete Structures and shown in Appendix A.

The design of flat slabs depends on dividing the slab into column strip including the inner columns line and field strip between each two column strips. The moments at these strips are calculated based on the building code of practice using custom nodes on Dynamo. The resulting moments are arranged in matrices as follows in Eqs. 7 and 8:

7 $$MC\, Distributed = \left[ {\begin{array}{*{20}c} {MC_{11} } & {MC_{12} } & \cdots & {MC_{15} } \\ {MC_{21} } & {MC_{22} } & \cdots & {MC_{25} } \\ \vdots & \vdots & \ddots & \vdots \\ {MC_{n1} } & {MC_{n2} } & \cdots & {MC_{n5} } \\ \end{array} } \right]$$

8 $$MF\, Distributed = \left[ {\begin{array}{*{20}c} {MF_{11} } & {MF_{12} } & \cdots & {MF_{15} } \\ {MF_{21} } & {MF_{22} } & \cdots & {MF_{25} } \\ \vdots & \vdots & \ddots & \vdots \\ {MF_{n1} } & {MF_{n2} } & \cdots & {MF_{n5} } \\ \end{array} } \right]$$

where n is the total number of span-divisions for the flat slab in each of the Column strip and Field strip. The maximum number of elements per row is 5 based on the 5 distribution factors used.

In total, 4 matrices are generated for each flat slab, namely: moment column strip in long direction, moment column strip in short direction, moment field strip in long direction and moment field strip in short direction. These 4 moment matrices are then used for calculating the overall required area of steel reinforcement. The required steel area (*A*_*s*_) per strip per m is calculated according to the followed building code and arrange in a matrix form as shown in Eqs. 9 and 10.

9 $$A_{s} CS = \left[ {\begin{array}{*{20}c} {AC_{11} } & {AC_{12} } & \cdots & {AC_{15} } \\ {AC_{21} } & {AC_{22} } & \cdots & {AC_{25} } \\ \vdots & \vdots & \ddots & \vdots \\ {AC_{n1} } & {AC_{n2} } & \cdots & {AC_{n5} } \\ \end{array} } \right]$$

10 $$A_{s} FS = \left[ {\begin{array}{*{20}c} {AF_{11} } & {AF_{12} } & \cdots & {AF_{15} } \\ {AF_{21} } & {AF_{22} } & \cdots & {AF_{25} } \\ \vdots & \vdots & \ddots & \vdots \\ {AF_{n1} } & {AF_{n2} } & \cdots & {AF_{n5} } \\ \end{array} } \right]$$

where n is the total number of span-divisions for the flat slab in each of the column strip and field strip. The maximum number of elements per row is 5 based on the 5 distribution factors used.

**Figure 7:**
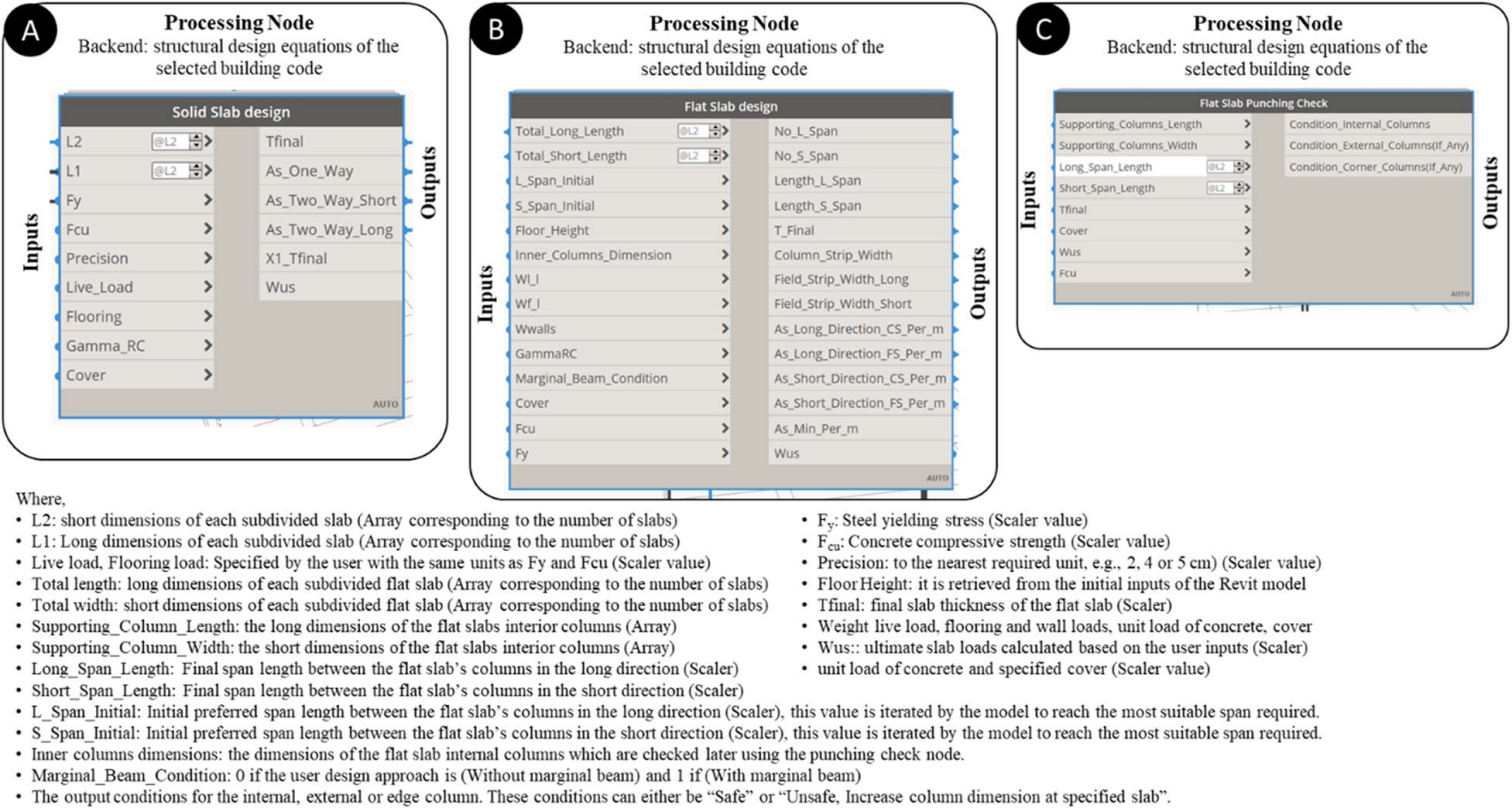
The developed Dynamo custom nodes for (**A**) solid slab, (**B**) flat slab design, (**C**) flat slab punching check.

The outputs of the slab design node are the final overall thickness of the slabs, required steel area for the solid slab or the flat slab, thickness per slab, and ultimate load of concrete slabs. Each group of the mentioned outputs represents one population for the optimization process. The reinforcement area steel for the slabs per meter run is calculated, then the model uses this area in determining the suitable steel diameter to be used. After selecting the required diameter corresponding to each calculated steel area, the model calculates the number of required bars per structural sub-slab. This data is used in determining the overall quantity of concrete and steel needed for the design of the proposed overall slab; the model transforms the total area of steel calculated into tonnage quantity using the density rule, and the total quantity of concrete is calculated in volume (m3). The user defines the current market price for steel and concrete and consequently the overall price of the solid slab can be calculated. The Flat Slab punching stress check must be done for the flat slab internal columns to ensure that each column can tolerate the applied load without punching the slab above it. The model checks the punching stress in the different types of columns as instructed by the ECP, then the design shear force is calculated. After calculating the design shear force, the model calculates the ultimate shear stress on the concrete. The critical shear section is compared with the concrete punching shear strength calculated by the used code of practice. All the previous steps are grouped in one custom node with the Flat Slab Punching check shown in Fig. 7 with the output conditions for the internal, external, or edge column. These conditions can either be "Safe", "Unsafe” or Increase column dimension at the specified slab."

### Automated design of beams (for solid slabs)

#### Load distribution

**Figure 8:**
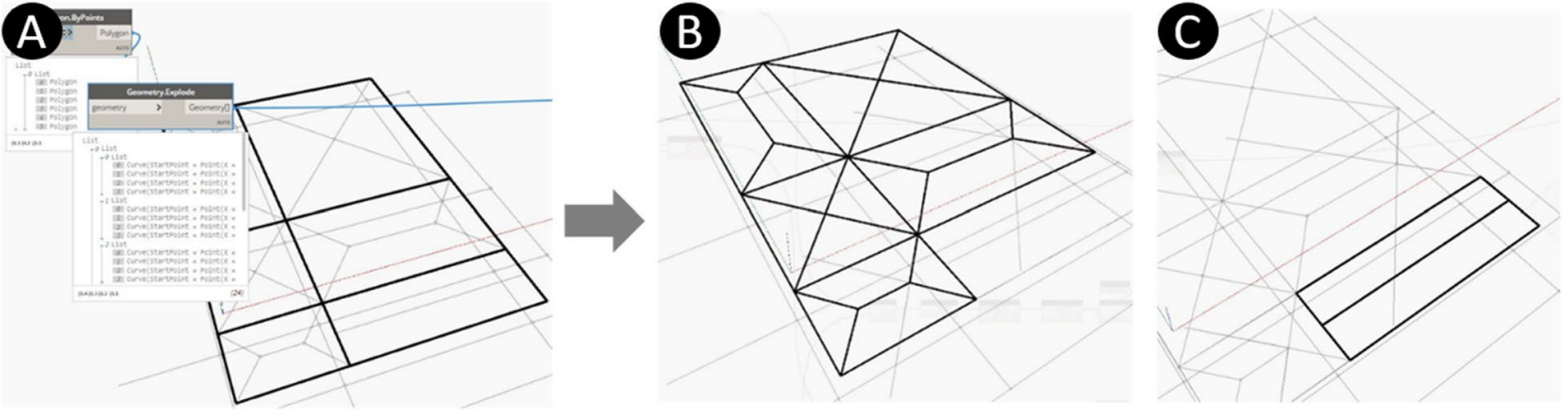
Load distribution of the floor slabs (**A**) Polygons representing slabs and curves representing beams, (**B**) Load distribution (two-way slab), (**C**) Load distribution (One-way slab).

The model is developed to use the quad points arrangement node in detecting the points representing the columns surrounding each sub-slab. Then each quad-points are joined into a polygon which, using a geometry explode function, can be divided into four joined curves as shown in Fig. 8A, representing the beams surrounding the slab and carrying its load. These curves are the inputs to the design of the concrete beams. The model detects the intersection points between the hypotenuses and joins them to create the trapezoid between two diagonals opposite to each other. The previous steps are done for all the two-way slabs of the model. The model is applied to lines between diagonals' intersection points parallel to the X-axis and those parallel to the Y-axis. The final load distribution of the two-way slabs is shown in Fig. 8B. After calculating the load distribution to the beams, the model transfers the triangular and the trapezoidal loads to distributed loads by a factor α, where α depends on the triangle/trapezoid height and the beam length as determined by the used building design code. For the one-way slabs, the long and short dimensions of the slab (long curves and short curves surrounding each slab) are detected. Then the short dimension is divided in half where the division points are detected; then, each opposite division point in a slab is joined; this represents the short-distance load division (Typical one-way slab). The final load distribution of the one-way slabs is shown in Fig. 8C.

#### Beam load grouping

**Figure 9:**
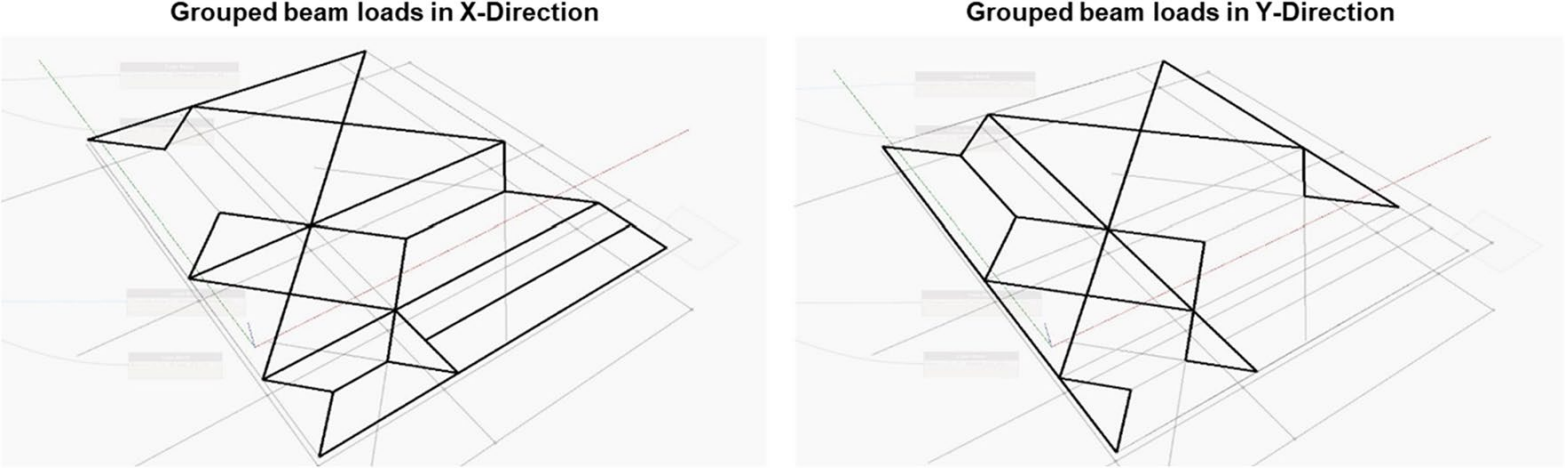
Grouped beam loads in the X and Y directions.

After determining the slab load distribution within each sub-divided slab, the model evaluates the load areas joined with one intersection curve. This intersection curve represents the beam holding the area loads from different sub-divided slabs. The slabs' grouped beam loads are shown in Fig. 9 in the X-Direction and the Y-Direction. These final two load distributions for the beams are presented in Eqs. 11 and 12 where each row represents one beam (One curve), and each column contains the poly curve areas carried by this beam.

11 $$BeamLoads_{X} = { }\left[ {\begin{array}{*{20}c} {PC_{11} } & {PC_{1m} } \\ {PC_{21} } & {PC_{2m} } \\ \vdots & \vdots \\ {PC_{n1} } & {PC_{nm} } \\ \end{array} } \right]$$

12 $$BeamLoads_{Y} = { }\left[ {\begin{array}{*{20}c} {PC_{11} } & {PC_{1m} } \\ {PC_{21} } & {PC_{2m} } \\ \vdots & \vdots \\ {PC_{n1} } & {PC_{nm} } \\ \end{array} } \right]$$

where n is the total number of beams in the plan and m is the number of loads carried by each beam (m can be 1 or 2; 2 in case of internal beams and 1 in case of external beams).

All the previous steps are grouped in one custom node with the name of Beams Load Areas with the outputs of the grouped load areas in the X and Y directions, and the intersecting curves with these load areas in the X and Y directions representing the beams.

#### Bending moment calculation for beams

Unlike the structural slabs, the empirical method for the design cannot be applied because most of its assumptions depend on the minor difference between the studied elements. However, in most cases there are significant differences between beams in terms of loads, dimensions, etc. Since beams are considered indeterminate structures, the Moment Distribution Method is used to calculate their bending moments since it is considered as one of the most reliable ways of doing so for indeterminate structures^24^. The model uses the C-J curve^25^ to calculate the required area of steel; this curve is based on the ultimate limit design procedure where the area of steel is calculated according to the applied moment. The final calculated area steel is compared to the minimum requirement based on the building design code. The curve is tabulated in the building code by incremental increase of 0.05 with minimum limit of 2.65 and maximum limit of 4.85 for the C value. The C-J matrix is presented in Eq. (13).

13 $$C - J\, points = \left[ {\begin{array}{*{20}c} {C_{11} } & {J_{12} } \\ {C_{21} } & {J_{22} } \\ \vdots & \vdots \\ {C_{n1} } & {J_{n2} } \\ \end{array} } \right]$$

The mathematical form of C_1_ and J are represented in the following Equation 14 and 15:

14 $$C_{1} = \sqrt {\frac{1}{{\left( \frac{2}{3} \right)*\left( {\frac{1}{{\lambda_{c} }}} \right)*0.8*\left( \frac{c}{d} \right)*(1 - 0.4*\left( \frac{c}{d} \right)}}}$$

15 $$J = \left( {\frac{1}{{\lambda_{s} }}} \right)*\left( {1 - 0.4*\left( \frac{c}{d} \right)} \right)$$

where ${\uplambda }_{{\text{c}}}$ is 1.5, c is compression region depth, d is clear depth above cover and ${\uplambda }_{{\text{s}}}$ is 1.15.

The derived moment loads for each slab in both the X and Y direction are used in calculating the value of C as presented in Eq. (16). The model uses the C-J points matrix to determine the value of J corresponding to the calculated value of C. then this J value is used in calculating the area of steel required (A_S_) as presented in Eq. (17). These procedures are done for the one and two-way slabs. The model rounds up the computed value of C to the nearest 0.05 to be able to detect the corresponding J value from the matrix.

16 $$C = {\raise0.7ex\hbox{$d$} \!\mathord{\left/ {\vphantom {d {\sqrt {\frac{{M_{u} }}{{f_{cu} *B}}} }}}\right.\kern-\nulldelimiterspace} \!\lower0.7ex\hbox{${\sqrt {\frac{{M_{u} }}{{f_{cu} *B}}} }$}}$$

17 $$A_{S} = {\raise0.7ex\hbox{${M_{u} }$} \!\mathord{\left/ {\vphantom {{M_{u} } {\left( {f_{y} *J*d} \right) }}}\right.\kern-\nulldelimiterspace} \!\lower0.7ex\hbox{${\left( {f_{y} *J*d} \right) }$}}$$

where d is the clear distance of the slab thickness (thickness- Cover specified by the code), f_cu_ is the concrete ultimate stress, B is the effective horizontal distance which in case of the slab = 1000 mm and f_y_ is the steel yielding stress.

A custom node for all previous steps for calculating steel area is created with the output of the steel required for supports, midspans, and in case there is only one beam. The model also applies shear structural design to the beams parallel to the X direction in one node and the beams parallel to the Y direction in another node. The outputs are areas of steel required for shear stirrups presented in Eq. (18) where each row represents an axis in the studied direction (X or Y), and each column represents a support on this axis.

18 $$AS_{s} = \left[ {\begin{array}{*{20}c} {AS_{11} } & {AS_{12} } & \cdots & {AS_{1m} } \\ {AS_{21} } & {AS_{22} } & \cdots & {AS_{2m} } \\ \vdots & \vdots & \ddots & \vdots \\ {AS_{n1} } & {AS_{n2} } & \cdots & {AS_{nm} } \\ \end{array} } \right]$$

where n is the total number of axes in the studied direction and m is the number of supports per axis.

#### Beam shear reinforcement calculation

Stirrups are essential reinforcements in beams to support against shear forces. The distributed load on the beam is calculated by Eq. (19):

19 $$Total\, Loads\, on\, Beam = O.w\, of\, beam + Slab\, Load + Wall\, Loads$$

where O.W is the own weight of the beam and is equal to (Beam cross section area * Unit load of concrete defined by the user).

The shear loads on each beam are used for the calculation of the beams shear load reactions. The model uses shear distribution method for the indeterminate structure to calculate the reactions. First, the direct shear for each beam is calculated using Eq. (20):

20 $$DIR. V = {\raise0.7ex\hbox{${W*L}$} \!\mathord{\left/ {\vphantom {{W*L} 2}}\right.\kern-\nulldelimiterspace} \!\lower0.7ex\hbox{$2$}}$$

where W is the total loads on beam and L is the Beam Length.

Since each beam is subject to a uniform distributed load, then the DIR.V. will be equal at the left and right supports of the beam.

Second, the auxiliary shear is calculated, which is the shear distributed to each support for the equilibrium state to be reached and it is driven from the results of the moment distribution previously implemented following Eq. (21):

21 $$AUX. V = {\raise0.7ex\hbox{${\sum MF_{i} }$} \!\mathord{\left/ {\vphantom {{\sum MF_{i} } {L_{i} }}}\right.\kern-\nulldelimiterspace} \!\lower0.7ex\hbox{${L_{i} }$}}$$

where MFi is the sum of the two bending moments at the right and left supports of each beam and L is the studied beam length.

The AUX.V will be equal at the left and right supports of the beam similar to the DIR.V.

Third, the total shear at each beam support will equal to the sum of direct shear and the auxiliary shear at this support. Then the final shear reactions per support will be given by Eq. (22):

22 $$V.Reaction = \left( {\sum DIR.V, AUX.V} \right)_{Left} + \left( {\sum DIR.V, AUX.V} \right)_{Right}$$

All the previous steps are grouped and coded in a custom node on Dynamo, with the reactions as outputs as presented in Eq. (23) where each row represents an axis in the studied direction (X or Y) and each column represents a support per this axis.

23 $$V.Reactions = \left[ {\begin{array}{*{20}c} {VR_{11} } & {VR_{12} } & \cdots & {VR_{1m} } \\ {VR_{21} } & {VR_{22} } & \cdots & {VR_{2m} } \\ \vdots & \vdots & \ddots & \vdots \\ {VR_{n1} } & {VR_{n2} } & \cdots & {VR_{nm} } \\ \end{array} } \right]$$

where n is the total number of axes in the studied direction and m is the number of supports per each axis.

After calculating the shear stresses, the steel area per linear meter of beam length is calculated in accordance with the equations developed according to building code and coded into a custom node on Dynamo. The calculated area of steel required for shear stirrups is presented in Eq. (24) where each row represents an axis in the studied direction (X or Y) and each column represents a support on this axis.

24 $$AS_{s} = \left[ {\begin{array}{*{20}c} {AS_{11} } & {AS_{12} } & \cdots & {AS_{1m} } \\ {AS_{21} } & {AS_{22} } & \cdots & {AS_{2m} } \\ \vdots & \vdots & \ddots & \vdots \\ {AS_{n1} } & {AS_{n2} } & \cdots & {AS_{nm} } \\ \end{array} } \right]$$

where n is the total number of axes in the studied direction and m is the number of supports per each axis.

Similar to the structural slabs, the model converts all the calculated reinforcement steel area for the beams into tonnage quantities using Eq. (25) and calculates quantity of concrete for the beams then calculates the overall market price for steel and concrete based on the unit price of each defined by the user.

25 $$AS_{Ton} = AS_{m} *7.850$$

where $AS_{Ton}$ is the calculated area of steel in Tons and $AS_{m}$ is the calculated area of steel in meters.

The tonnages of steel and volumes of concrete of the slabs and beams are summed together for the preparation of different objective functions presented in the following sections. These objective functions are the required functions for the optimization process. One of the advantages of using the visual programming language in building this model is the generic nature, the capability of the model to be applied on different plans arrangement with different spaces and dimensions.

## Optimization module

**Figure 10:**
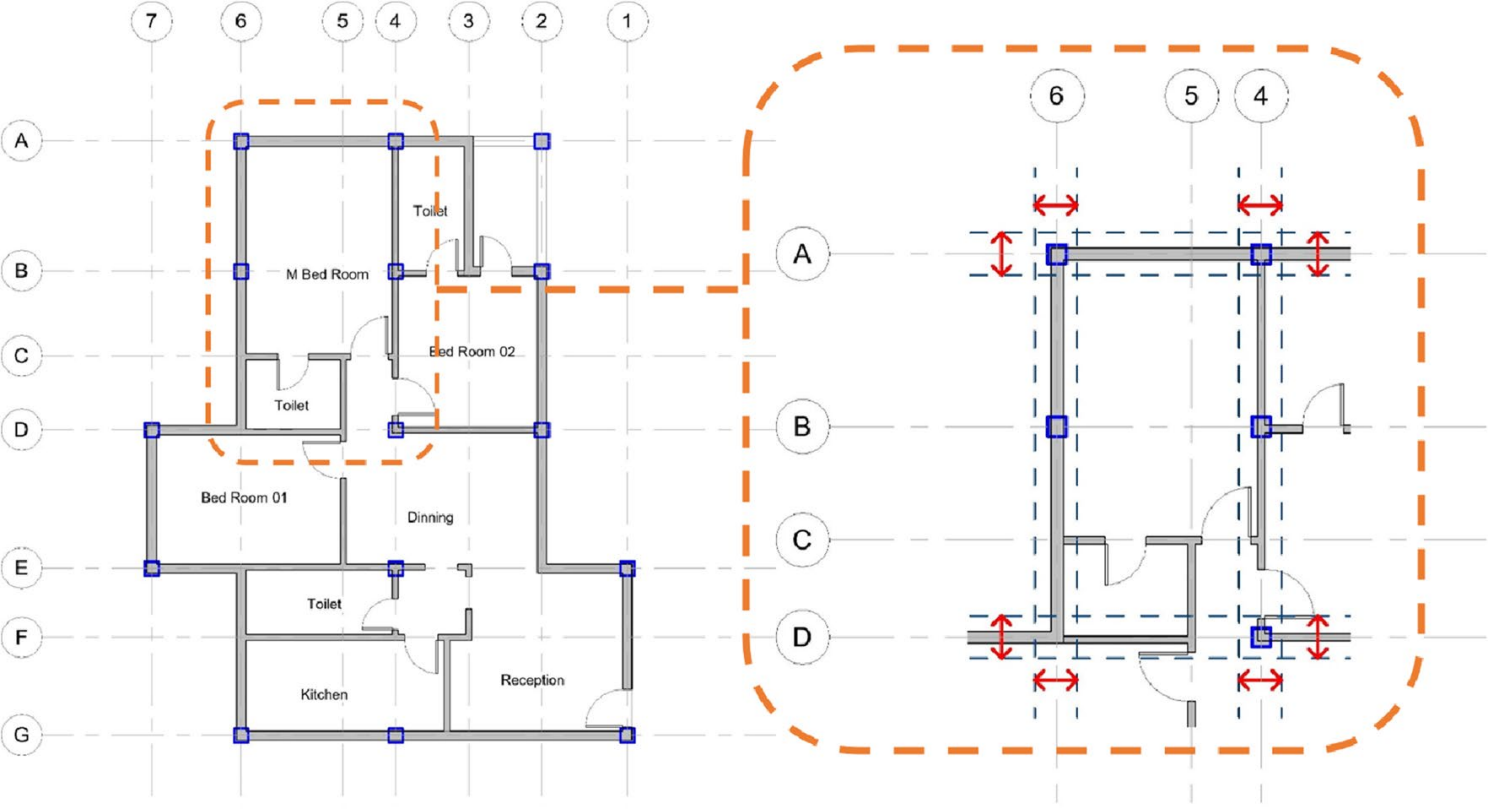
Demonstrating the dimension boundaries concept.

The model variables are identified as upper and lower limits of each space in the architectural plan; the architect determines these limits depending on the functionality requirements. For example, a certain space on the plan will have an X and Y dimensions. Each of them will have an upper and lower limit considering that the increase in a certain dimension on the plan will subsequently decrease the adjoining dimension. These possible changes in the dimensions based on the architectural limits create the window for the optimization model with the objective function of minimizing the cost of the structural elements with the maximum utilization of the available architectural spaces. Figure 10 demonstrates this concept of the upper and lower boundaries for each dimension, providing a window for the numerous numbers of iterations in the optimization model.

Another important constraint is the overall dimensions in the X and Y dimension (L1 and L2) which are constant variables also defined by the user for best utilization of the available space.

The developed model makes use of the Optimo optimizer tool that is easily integrated with the model's BIM environment. The Optimo optimizer utilizes genetic algorithms (GA) for the optimization process. The genetic algorithm has significant advantages that has been used in different BIM optimization models in the past such as: parallelism, requirement of less information than the typical mathematical models and equations, providing multiple optimal solutions with less complex analytical form, and solving large problems with higher variables and constraints^26–28^. In the context of this research, the population is the range of different possible dimensions provided from the architectural design in the given optimization framework.

The first step of the optimization process is defining the initial list of random variables with their constraints. As mentioned before, the model's variables are the plan dimensions in the X and Y directions. Each of these variable dimensions has upper and lower limit constraints defined by the architect for the functionality of the space. The user defines a row vector matrix for the lower and upper limits for the dimensions in the X and Y directions, as shown in Eq. (26). A mathematical node is used to develop the random variables matrix needed. The inputs to this node is the row vector matrices of the upper and lower limits for the dimensions, the number of random population size required for the optimization process defined by the user, and the seed number, which is a random number between 0 and 1. This random generating number node is applied to the limits in the X and Y direction.

26 $$Variables_{i} Limits_{j} = \left[ {\begin{array}{*{20}c} {LI_{11} } & {LI_{12} } & \cdots & {LI_{1m} } \\ \end{array} } \right]_{ij}$$

where m is the number of dimensions in the studied X/ Y Directions, i is (Upper or Lower), and j is (X or Y Direction).

The results of the previous random number generating node are shown in Eq. (27):

27 $$Dimensions\,Random\,Variables_{i} = { }\left[ {\begin{array}{*{20}c} {RN_{11} } & {RN_{12} } & \cdots & {RN_{1m} } \\ {RN_{21} } & {RN_{22} } & \cdots & {RN_{2m} } \\ \vdots & \vdots & \ddots & \vdots \\ {RN_{n1} } & {RN_{n2} } & \cdots & {RN_{nm} } \\ \end{array} } \right]$$

where i is the studied X or Y direction, n is the total number of dimensions in the studied direction and m is the required size of the population.

The matrix of derived random variables needs to follow the overall higher constraint dimensions (L1 and L2); the random numbers generated per population are summed together then remapped by Eq. (28) to create a population of random variables between the required upper and lower limits. Their summation follows the higher constraint limits of the constant overall dimensions in the X and Y directions.

28 $$Remapped\,RN_{nm} = { }\frac{{RN_{nm} }}{{\sum RN_{nm} \,per\,population\,per\,Direction_{i} }}{*}L_{i}$$

where ${\text{RN}}_{{{\text{nm}}}}$ The random dimensions variables and i is the overall dimension in X or Y direction.

All the previous steps are grouped in one custom node with the name of “General Random Dimensions” Variables with: the inputs of the row vector matrices of the upper and lower limits for the dimensions in the X and Y directions; the precision level required; the overall constant dimensions in the X and Y directions; the number of random population size defined by the user and the number of required objectives (For example, 1 for a single objective, 2 for two objectives, etc. The defined meaning of precision is the degree to which the user wants to change the dimension's units; if the precision is 1, the decimal digits are 1, which is equivalent to working with the Decimeters unit. If precision is 2, the decimal digits are 2, equivalent to the Centimeters unit. Three is equivalent to a millimeters unit; this provides a wider range of generic use for the user. The X and Y random lists are grouped using a transpose function node to create one random generating list. This custom node creates a sub-list of zeros in its end, equal to the number of required objectives of the optimization process. This Zero list assigns the objective values derived from the assigned objective fitness function. The General Random Dimensions Variables node is presented in the following Eq. (29).

29 $$Optimization\, Random\, Variables = \left[ {\begin{array}{*{20}c} {RV_{11} } & {RV_{12} } & \cdots & {RV_{1m} } \\ {RV_{21} } & {RV_{22} } & \cdots & {RV_{2m} } \\ \vdots & \vdots & \ddots & \vdots \\ {RV_{n1} } & {RV_{n2} } & \cdots & {RV_{nm} } \\ 0 & 0 & \cdots & {0_{{\left( {n + 1} \right)m}} } \\ \end{array} } \right]$$

where n is the total number of dimensions in both X and Y directions, where each RV is between the Upper and Lower Limits specified, and m is the required size of the population.

After defining the random constrained variables, the model assigns the objective structure–function to the list of zeros defined at the end of the random variables. The model takes each population of random variables, enters them in the objective functions, and then assigns the results to the zero-matrix defined for all the required iterations. The previous steps represent the initial population for the optimization process; then, the model uses the non-dominated Sorting genetic algorithm (NSGA-II) containing a generation algorithm function for applying the genetic optimization. The generated algorithm considers the initial population determined as the parent solutions then implement the mutation and crossover processes for the generation of children solutions. The NSGA-II custom node contains a sorting node that uses the Pareto front technique for arranging the populations' results. The user specifies the required number of iterations, which increases the accuracy (Precision) of the optimization process if increased. The optimization process takes place until a near-optimum solution is reached, and all the input populations give the same constant optimum result. The final optimum solution node presents the optimum dimensions considering all the limits and the optimum result. The results of the previous iterations are shown in Eq. (30) after the optimum solutions have been fixed.

30 $$Optim. Results = \left[ {\begin{array}{*{20}c} {FD_{11} } & {FD_{12} } & \cdots & {FD_{1m} } \\ {FD_{21} } & {FD_{22} } & \cdots & {FD_{2m} } \\ \vdots & \vdots & \ddots & \vdots \\ {FD_{n1} } & {FD_{n2} } & \cdots & {FD_{nm} } \\ {OR_{{\left( {n + 1} \right)1}} } & {OR_{{\left( {n + 1} \right)2}} } & \cdots & {0R_{{\left( {n + 1} \right)m}} } \\ {OR_{{\left( {n + 2} \right)1}} } & {OR_{{\left( {n + 2} \right)2}} } & \cdots & {OR_{{\left( {n + 2} \right)m}} } \\ \vdots & \vdots & \ddots & \vdots \\ {OR_{{\left( {n + i} \right)1}} } & {OR_{{\left( {n + i} \right)2}} } & \cdots & {OR_{{\left( {n + i} \right)m}} } \\ \end{array} } \right]$$

where FD is final optimum dimensions OR is the Final corresponding optimum objective Function results, n is the total number of dimensions in both X/ Y directions, m is the population's required size, and i is the number of objectives required.

There could be only one objective function in the optimization process. This objective function could be (1) minimize the total cost of steel and concrete, (2) minimize the concrete quantity, or (3) minimize the steel quantity. Table 1 presents these objective functions for the different structural systems and the corresponding results that are within the capabilities of the developed model. The developed model is flexible to address any of those objective functions based on the user’s preference. The model is designed to provide flexibility to users. For example, if a user has an architectural design with no preference for the structural system, the model can run multiple scenarios and select the optimum structural system and the optimum design in that structural system as shown in the first row of Table 1. If the user has a preference for the type of structural system (solid or flat slab), the model is able to abide by the user's preference and run the optimization module on just the structural system selected by the user, if not, the model can mitigate between the different types to expand the population and reach the most suitable and optimum design type as well as the orientation.

**Table 1:** Objective functions by the developed model.

Objective	Valid for	Mathematical representation	Output
**Default configuration**			
Minimize total cost of concrete and steel	Architectural plans where the user has no preference on the slab system	$\min \sum C_{SS} \left( {\left( {X_{Q} *R_{C} } \right) + \left( {Y_{Q} *R_{S} } \right) } \right) + C_{FS} \left( {\left( {X_{Q} *R_{C} } \right) + \left( {Y_{Q} *R_{S} } \right) } \right) + C_{B} \left( {\left( {X_{Q} *R_{C} } \right) + \left( {Y_{Q} *R_{S} } \right) } \right)$	Selection of optimum slab system Optimal structural design of that system (concrete dimensions + steel area) Cost and quantity takeoff of the resulting design
**Other configurations where user has preset preferences for selection of slab system**			
Minimize cost of the flat slab system	Flat slab systems	$\min \sum C_{FS} \left( {\left( {X_{Q} *R_{C} } \right) + \left( {Y_{Q} *R_{S} } \right) } \right) + C_{B} \left( {\left( {X_{Q} *R_{C} } \right) + \left( {Y_{Q} *R_{S} } \right) } \right)$	Optimal structural design of the flat slab system (concrete dimensions + steel area) Cost and quantity takeoff of the resulting design
Minimize concrete quantity of flat slab		$\min \sum C_{FS} \left( {X_{Q} *R_{C} } \right) + C_{B} \left( {X_{Q} *R_{C} } \right)$	
Minimize steel quantity of flat slab		$\min \sum C_{FS} \left( {Y_{Q} *R_{S} } \right) + C_{B} \left( {Y_{Q} *R_{S} } \right)$	
Minimize cost of solid slab	Solid slab systems	$\min \sum C_{SS} \left( {\left( {X_{Q} *R_{C} } \right) + \left( {Y_{Q} *R_{S} } \right) } \right) + C_{B} \left( {\left( {X_{Q} *R_{C} } \right) + \left( {Y_{Q} *R_{S} } \right) } \right)$	Optimal structural design of the solid slab system (concrete dimensions + steel area) Cost and quantity takeoff of the resulting design
Minimize concrete quantity of solid slab		$\min \sum C_{SS} \left( {X_{Q} *R_{C} } \right) + C_{B} \left( {X_{Q} *R_{C} } \right)$	
Minimize steel quantity of solid slab		$\min \sum C_{SS} \left( {Y_{Q} *R_{S} } \right) + C_{B} \left( {Y_{Q} *R_{S} } \right)$	
Minimize cost of slabs	The plan has both solid and flat slabs in the same floor	$\min \sum C_{SS} \left( {\left( {X_{Q} *R_{C} } \right) + \left( {Y_{Q} *R_{S} } \right) } \right) + C_{FS}^{*} \left( {\left( {X_{Q} *R_{C} } \right) + \left( {Y_{Q} *R_{S} } \right) } \right) + C_{B} \left( {\left( {X_{Q} *R_{C} } \right) + \left( {Y_{Q} *R_{S} } \right) } \right)$	Optimal structural design of the hybrid flat & solid slab system (concrete dimensions + steel area) Cost and quantity takeoff of the resulting design
Minimize concrete quantity of slabs		$\min \sum C_{SS} \left( {X_{Q} *R_{C} } \right) + C_{FS}^{*} \left( {X_{Q} *R_{C} } \right) + C_{B} \left( {X_{Q} *R_{C} } \right)$	
Minimize steel quantity of slabs		$\min \sum C_{SS} \left( {Y_{Q} *R_{S} } \right) + C_{FS}^{*} \left( {Y_{Q} *R_{S} } \right) + C_{B} \left( {Y_{Q} *R_{S} } \right)$	

where $C_{SS}$ Cost of solid slabs which is a function of quantity and unit rate; $C_{FS}$ Cost of Flat slabs which is a function of quantity and unit rate; $C_{B}$ Cost of Beams which is a function of quantity and unit rate; $X_{Q}$ Total quantity of reinforcement concrete material; $R_{C}$ Price Unit rate of reinforcement concrete material; $Y_{Q}$ Total quantity of reinforcement steel material; $R_{S}$ Price Unit rate of reinforcement steel material.

*Indicator that the flat slabs location is determined by the user instead of the model determination.

## Case studies

The proposed optimization model was implemented on 11 case studies different of number of spaces, dimensions arrangement in X/Y direction, types of structural slabs, and concrete properties. In other words, the differences between the case studies were in the orientations, quantities, functions, levels, etc. This implementation on different buildings to evaluate the model’s capabilities in optimizing designs and minimizing costs. Each case study is a real concrete building that is already designed by structural engineers and approved for construction. Some of the buildings in the case studies are already constructed.

**Figure 11:**
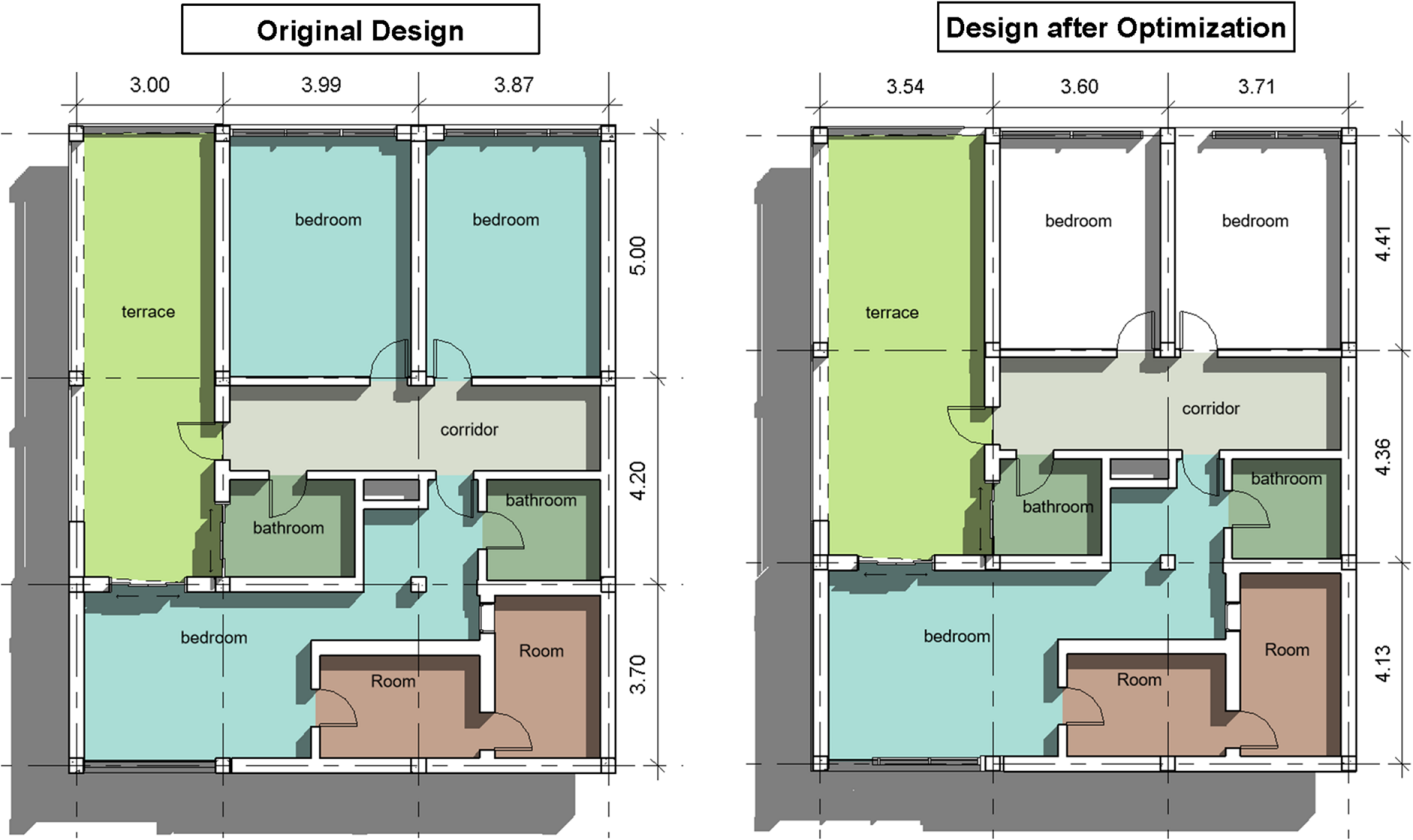
Case study #9.

The building in the first case study is a 2-story residential villa. The structural system was designed to be solid slabs with 16 concrete columns. The architectural design of the building and the other parameters were inputted in the developed model, and the optimization engine ran with a population size and specified number of iterations. The original design had initial grid dimensions in X-Direction of 3.0, 3.99 and 3.87 m, and initial grid dimensions in Y-Direction of 3.7, 4.2, 5.0 m as shown in Fig. 11. The concrete and steel cost of the original design was EGP 63,110 per floor. An optimization scenario was executed having an objective function to minimize the total concrete and steel cost while limiting the design to solid slabs. The cost after optimization is 55.640 EGP per floor, which is considered a 11.84% saving in cost.

Table 2 presents a summary of the case studies before and after optimizing using the developed model. It can be observed that all of the 11 case studies witnessed savings in the material cost ranging from 3.21% to as high as 13.5%. The average savings is 8.77%. The Table also presents the proposed upper and lower limit for each floor dimension.

**Table 2:** Optimized cost and % savings for the analyzed case studies.

		Grid dimension of original design (m)							Grid dimension after optimization (m)							Concrete quantity (m3)			Steel quantity (kg)			Material cost per floor (EGP)		
Case-study *#*	Direction	D1	D2	D3	D4	D5	D6	D7	D1	D2	D3	D4	D5	D6	D7	Original	After optimizat ion	Savings (%)	Original	After optimizat ion	Savings (%)	Original	After optimizat ion	Savings (%)
1	X-Direction	3.14	3.20						3.12	3.22						24.3	23.3	4.12	393	403	–	52,527	50,625	3.62
	Y-Direction	3.38	8.88	4.90					4.00	8.20	4.97													
2	X-Direction	3.60	4.00	4.40	2.60	3.20			3.60	4.20	4.20	2.60	3.20			21.1	20.1	4.98	376	375	0.3	45,960	43,850	4.59
	Y-Direction	2.10	2.70	2.10					2.30	2.30	2.30													
3	X-Direction	3.50	3.50	5.70					3.58	3.58	5.53					29.5	28.5	3.39	583	575	1.4	64,830	62,750	3.21
	Y-Direction	3.10	4.00	3.76					3.04	3.92	3.90													
4	X-Direction	3.00	3.00	3.73					3.06	3.06	3.61					30.3	28.6	5.61	575	575	–	66,350	62,950	5.12
	Y-Direction	3.40	3.00	3.50	4.00	3.16			3.40	3.10	3.48	3.86	3.22											
5	X-Direction	6.30	5.00						6.20	5.10						45.2	38.3	15.27	939	972	–	99,790	86,320	13.50
	Y-Direction	5.00	4.00	3.00	4.00				4.50	4.50	3.50	3.50												
6	X-Direction	3.00	3.50	2.80	3.00				3.00	3.15	3.00	3.15				45	39.4	12.44	907	1005	–	99,070	88,850	10.32
	Y-Direction	5.00	3.50	3.20	3.30	3.00	2.80		3.83	3.90	3.43	3.32	3.25	3.07										
7	X-Direction	3.00	3.50						2.88	3.62						17.7	16.15	8.76	344	341	0.9	38,840	35,710	8.06
	Y-Direction	3.50	3.60	2.50	4.50				3.49	3.69	2.05	4.87												
8	X-Direction	4.00	3.30						3.90	3.40						54.3	47.8	11.97	1070	1031	3.6	119,300	105,910	11.22
	Y-Direction	6.00	6.50	4.50	5.00	3.00	3.00	3.10	5.60	5.90	4.50	4.50	3.70	3.40	3.50									
9	X-Direction	3.87	3.99	3.00					3.71	3.60	3.54					28.6	24.5	14.34	591	664	–	63,110	55,640	11.84
	Y-Direction	3.70	4.20	5.00					4.13	4.36	4.41													
10	X-Direction	4.00	3.00						4.14	2.86						63.1	54.8	13.15	1066	1021	4.2	136,860	119,810	12.46
	Y-Direction	5.00	4.40	5.50	7.80	4.50	4.00	2.10	5.05	4.30	5.65	8.40	4.45	3.85	1.60									
11	X-Direction	3.00	3.00	3.00					3.00	3.00	3.00					33.7	29.6	12.17	664	558	16.0	74,040	64,780	12.51
	Y-Direction	3.30	3.80	2.70	2.30	5.10	1.60		3.30	4.28	2.15	1.37	6.70	1.00										

**Figure 12:**
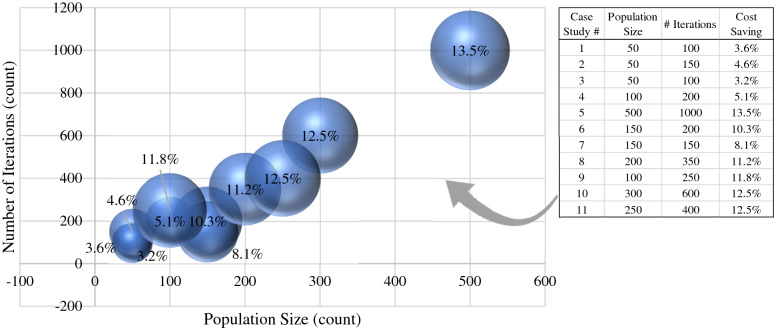
Sensitivity of the cost saving (bubble size) to the population size and number of iterations.

To determine the impact of different model’s factors, a small sensitivity analysis was conducted where different population size and number of iterations were used in the 11 case studies. the following graphs of the analyzed case studies were concluded. The population sizes of the various optimization processes were arranged in ascending order and plotted vs. the cost-saving percentages as shown in Fig. 12. As the population size of the optimization process increases, the model can evaluate more random variables from the population and reach a more accurate optimum result. The trend shown in the graph presents this conclusion with a directly exponential relationship between the population size and cost-saving percentage.

**Figure 13:**
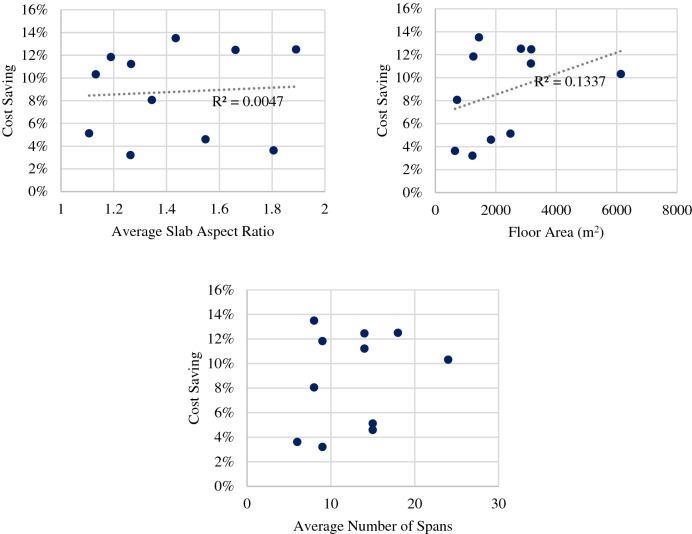
Sensitivity of the cost saving to the average slab aspect ratio, total floor area, and average number of spans.

Another sensitivity analysis was conducted to see whether the aspect ratio of the slabs (the gridline) affects the resulting savings. Average aspect ratio for each case study was calculated as follows: The aspect ratio of each slab within the grid is calculated (so, if we have a 3 by 3 grid, there would be 9 slabs), then the average of all these is considered. An aspect ratio of 1 means that the slabs are square shaped. A higher number indicates a more rectangular shape. As shown in Fig. 13, it was found that there is no strong relation between the aspect ratio and the corresponding savings. This means that the model is efficient in most traditional aspect ratios and able to result in cost savings. The same analysis was done for the relation of cost saving with the floor area and avg. number of spans giving the same observation, this refers to the higher complex ability of the model due to the large number of different factors inside the design process as well as the different stages undergo by the model.

Through the analysis of the automated design- optimization model and the results of the case studies, this research presents a relevant contribution to the BIM 9D lean construction; to deliver and manage progress of a project more efficiently while making the best use of resources and capital, this dimension places a strong emphasis on resource management techniques. These techniques aim to improve the allocation and use of materials, labor, equipment, and tools throughout the project’s duration. All resources used in the construction and running of a project are analyzed by 9D BIM. For instance, helpful information can be gleaned regarding the most effective use of trucks for the transportation of materials, the reduction of onsite trucks and circulation routes, the elimination of monotonous non-value adding activities, and the shortening of cycle times^29^.

## Conclusion

This research outlines a model that integrates the rectilinear architectural design, the structural design, and the corresponding cost required for concrete floors. The approach is for optimizing the architectural and structural design integration process to reach optimum structural elements' cost while considering the functionality of the architectural design and cost savings in the structural design. The proposed model was developed using parametric constraint-based modeling in BIM as an automated design method. A spatial Architecture- Structural elements genetic optimization model was developed that included several developed algorithms. The algorithms were coded in a BIM environment using design script visual programming language, where Autodesk Revit was used as the BIM environment and Dynamo add-in as design script compiler in Autodesk Revit. The model was tested on 11 different case studies where the model achieved more than 15% cost saving per floor. The case studies differed in structural systems, space functionality, and dimensions orientations.

The model can handle the regular shaped floors in this stage, it is recommended to further enhance the model to handle the irregular floors. Future research will develop a user-friendly interface for the model; right now, the user needs to modify the objective function custom node and population generation custom node to allow for different dimensions orientations as inputs of different models. There is still a window for designing other structural elements like other types of slabs, columns, and foundations. Designing of more complicated structural components such as foundations, shear walls, etc. is applicable through similar model formulation, coding and using the same optimization engine which can be an opportunity for future research and development.

## Supplementary Information

Supplementary Information.

## Data Availability

The datasets used and/or analyzed during the current study are available from the corresponding author on reasonable request.
