# Supplementary material for: Automated BIM-based structural design and cost optimization model for reinforced concrete buildings
Source: Sci Rep. 2022 Dec 14;12:21616. doi: 10.1038/s41598-022-26146-6 (PMC9751273; doi:10.1038/s41598-022-26146-6)
Supplement: Supplementary file 1 — Supplementary Information. [file 41598_2022_26146_MOESM1_ESM.docx]

**Automated BIM-Based Structural Design and Cost Optimization model for Reinforced Concrete Buildings**

# Appendix

|  | | ${GC}_{X}= \left[ \begin{matrix} X_{11} & X_{12} & \cdots& X_{1m} \end{matrix} \right]$ | | | | ${GC}_{Y}= \left[ \begin{matrix} Y_{21} & Y_{22} & \cdots& Y_{1n} \end{matrix} \right]$ | | [1] |
| --- | --- | --- | --- | --- | --- | --- | --- | --- |
| where *m* is the number of Grid Lines in X direction and n the number of Grid Lines in Y direction. | | | | | | | | |
|  | $Dim X= \left[ \begin{matrix} X_{11} \\ X_{21} \\ \vdots\\ X_{m1} \end{matrix} \right]$ | | | $Dim Y= \left[ \begin{matrix} Y_{11} \\ Y_{21} \\ \vdots\\ Y_{n1} \end{matrix} \right]$ | | | [2], [3] | |
| where *m* is the number of Variables in X direction and n the number of Variables in Y direction. | | | | | | | | |
|  | | $IP= \left[ \begin{matrix} I_{11} & I_{12} & \cdots& I_{1m} \\ I_{21} & I_{22} & \cdots& I_{1m} \\ \vdots& \vdots& \ddots& \vdots\\ I_{n1} & I_{n2} & \cdots& I_{nm} \end{matrix} \right]$ | | | | | | [4] |
| where n is the number of Gird Lines and m is the number of Intersection points per Line. | | | | | | | | |
|  | | $LV= \left[ \begin{matrix} L_{11} & L_{12} & \cdots& L_{1m} \\ L_{21} & L_{22} & \cdots& L_{2m} \\ \vdots& \vdots& \ddots& \vdots\\ L_{n1} & L_{n2} & \cdots& L_{nm} \end{matrix} \right]$ | | | | | | [5] |
| where n is the number of model levels and m is the number of Intersection points per level. | | | | | | | | |
|  | | $OD= \left[ \begin{matrix} O_{11} & O_{12} & \cdots& O_{1m} \\ O_{21} & O_{22} & \cdots& O_{2m} \\ O_{31} & O_{32} & \cdots& O_{3m} \\ O_{41} & O_{42} & \cdots& O_{4m} \end{matrix} \right]$ | | | | | | [6] |
| where m is the number of Curves on the same side of the outer Dimension of the overall slab. | | | | | | | | |
|  | | | $Local U= \left[ \begin{matrix} 0 \\ U_{21} \\ \vdots\\ U_{m1} \\ 1 \end{matrix} \right]$ | | $Local V= \left[ \begin{matrix} 0 \\ V_{21} \\ \vdots\\ V_{n1} \\ 1 \end{matrix} \right]$ | | [7], [8] | |
| where *m* is the number of cumulative variables in U local direction and n the number of cumulative variables in V location direction, all the variables are between 0 and 1. | | | | | | | | |
|  | | $SP= \left[ \begin{matrix} {SP}_{11} & {SP}_{12} & \cdots& {SP}_{1m} \\ {SP}_{21} & {SP}_{22} & \cdots& {SP}_{2m} \\ \vdots& \vdots& \ddots& \vdots\\ {SP}_{n1} & {SP}_{n2} & \cdots& {SP}_{nm} \end{matrix} \right]$ | | | | | | [9] |
| where n is the number of column-lines in the Grid System and m is the number of columns location points per line. | | | | | | | | |

|  | | | | | $Quad Points= \left[ \begin{matrix} P_{11} & P_{12} & \cdots& P_{14} \\ P_{21} & P_{22} & \cdots& P_{24} \\ \vdots& \vdots& \ddots& \vdots\\ P_{n1} & P_{n2} & \cdots& P_{n4} \end{matrix} \right]$ | | | | | | | | | | | | | | | | | | | | | | | | | [10] | | | |  |
| --- | --- | --- | --- | --- | --- | --- | --- | --- | --- | --- | --- | --- | --- | --- | --- | --- | --- | --- | --- | --- | --- | --- | --- | --- | --- | --- | --- | --- | --- | --- | --- | --- | --- | --- |
| where n is the number of subdivided quadratic slabs in the overall proposed plan. | | | | | | | | | | | | | | | | | | | | | | | | | | | | | | | | | |  |
|  | | | | | | $Recommended Slab Thickness=\left. \begin{aligned} \frac{a \left( 0.85+ \left( \frac{F_{y}}{1600} \right) \right)}{15+ \left( \frac{20}{\left( \frac{b}{a} \right)} \right)+\left( 10\beta\right)}, &r\leq2 \end{aligned} \right.$ | | | | | | | | | | | | | | | | | | | [11] | | | | | |  |  |  |  |
| where a is the short dimension of the slab, b is the long dimension, Fy is the yielding stress of the steel (two-way slab) and $\beta$ is the ratio of the total length of continuous edges of the slab to the total perimeter. | | | | | | | | | | | | | | | | | | | | | | | | | | | | | | |  |  |  |  |
|  | | | | | | $Recommended S. Thickness=\left\{ \begin{aligned} \frac{L}{25}, &r>2 , Simple \\ \frac{L}{30}, &r>2 , Continuous One Side \\ \frac{L}{36}, &r>2 , Continuous Both Sides \\ \frac{L}{10}, &r>2 , Cantilever \end{aligned} \right.$ | | | | | | | | | | | | | | | | | | | [12] | | | | | |  |  |  |  |
| where L is the slab side Dimension (One-way slab). | | | | | | | | | | | | | | | | | | | | | | | | | | | | | | |  |  |  |  |
|  | | | | | $Wus= \left( 1.4*Dead Loads \right)+ \left( 1.6*Live Loads \right)\ldots\ldots\ldots\frac{kN}{m^{2}}$ | | | | | | | | | | | | | | | | | | | | | | | | | [13] | | | |  |
| where the Dead loads are the weights of concrete and flooring. and the Live loads are the moving loads as specified by the user. | | | | | | | | | | | | | | | | | | | | | | | | | | | | | | | | | |  |
|  | $\alpha=\left( 0.5*\left( r \right) \right)-0.15$ | | | | | | | | | | | | | | | | $\beta= \frac{0.35}{\left( r \right)^{2}}$ | | | | | [14], [15] | | | | | | | | |  |  |  |  |
| where *r equals* $\frac{m_{1}* L_{1}}{m_{2}* L_{2}}$*, m_1_ and m_2_ are distribution factors based on simple (1), continuous from one side (0.87) or continuous from both sides (0.76). L_1_ is the long dimension and L_2_ is the short dimension of the slab. r is between 1~2. If r is less than 1, the reciprocal is used and if r is more than 1,* $\alpha$ *equals 1 and* $\beta$ *equals 0.* | | | | | | | | | | | | | | | | | | | | | | | | | | | | | | |  |  |  |  |
|  | | | | | | | | | | $C-J points= \left[ \begin{matrix} C_{11} & J_{12} \\ C_{21} & J_{22} \\ \vdots& \vdots\\ C_{n1} & J_{n2} \end{matrix} \right]$ | | | | | | | | | | | | | | | [16] | | | | | |  |  |  |  |
| where n is the total number of points extracted from the curve (Total 45 points). | | | | | | | | | | | | | | | | | | | | | | | | | | | | | | |  |  |  |  |
|  | | $C_{1}= \sqrt{\frac{1}{\left( \frac{2}{3} \right)*\left( \frac{1}{\lambda_{c}} \right)*0.8*\left( \frac{c}{d} \right)*(1-0.4*\left( \frac{c}{d} \right)}}$ | | | | | | | | | | | | | | | | $J= \left( \frac{1}{\lambda_{s}} \right)*\left( 1-0.4*\left( \frac{c}{d} \right) \right)$ | | | | | | [17], [18] | | | | | | |  |  |  |  |
| where $\lambda_{c}$ *is 1.5, c is compression region depth, d is clear depth above cover and* $\lambda_{s}$ *is 1.15.* | | | | | | | | | | | | | | | | | | | | | | | | | | | | | | |  |  |  |  |
|  | | | | | | | | $C= \frac{d}{\sqrt{\frac{M_{u}}{f_{cu}*B}}}$ | | | | | | | | $A_{S}= \frac{M_{u}}{\left( f_{y}*J*d \right)}$ | | | | | | | | | [19], [20] | | | | | | | |  |  |
| where *d is the clear distance of the slab thickness (thickness- Cover specified by the code), f_cu_ is the concrete ultimate stress, B is the effective horizontal distance which in case of the slab = 1000 mm and f_y_ is the steel yielding stress.* | | | | | | | | | | | | | | | | | | | | | | | | | | | | | | | | |  |  |
|  | | | | | | $Slab Thickness=Larger of\left\{ \begin{aligned} 150, &minimum safe depth (mm) \\ \frac{L}{32}, &Without Drop panels \\ \frac{L}{36}, &With Drop panels \end{aligned} \right.$ | | | | | | | | | | | | | | | | | | | [21] | | | | | |  |  |  |  |
| where L is the maximum span length specified. | | | | | | | | | | | | | | | | | | | | | | | | | | | | | | |  |  |  |  |
|  | | | | | | $M_{o}=\left. \begin{aligned} \frac{W_{u}* L_{2}}{8}* \left( L_{1}- \frac{2*D}{3} \right)^{2}, &Long Direction \end{aligned} \right.$ | | | | | | | | | | | | | | | | | | | [22] | | | | | |  |  |  |  |
|  | | | | | | $M_{o}=\left. \begin{aligned} \frac{W_{u}* L_{1}}{8}* \left( L_{2}- \frac{2*D}{3} \right)^{2}, &Short Direction \end{aligned} \right.$ | | | | | | | | | | | | | | | | | | | [23] | | | | | |  |  |  |  |
| where L_2_ is the short span, L_1_ is the Long span and D is the inner columns dimensions. | | | | | | | | | | | | | | | | | | | | | | | | | | | | | | |  |  |  |  |
|  | | | | | | $MC Distributed= \left[ \begin{matrix} {MC}_{11} & {MC}_{12} & \cdots& {MC}_{15} \\ {MC}_{21} & {MC}_{22} & \cdots& {MC}_{25} \\ \vdots& \vdots& \ddots& \vdots\\ {MC}_{n1} & {MC}_{n2} & \cdots& {MC}_{n5} \end{matrix} \right]$ | | | | | | | | | | | | | | | | | | | [24] | | | | | |  |  |  |  |
|  | | | | | | $MF Distributed= \left[ \begin{matrix} {MF}_{11} & {MF}_{12} & \cdots& {MF}_{15} \\ {MF}_{21} & {MF}_{22} & \cdots& {MF}_{25} \\ \vdots& \vdots& \ddots& \vdots\\ {MF}_{n1} & {MF}_{n2} & \cdots& {MF}_{n5} \end{matrix} \right]$ | | | | | | | | | | | | | | | | | | | [25] | | | | | |  |  |  |  |
| where n is the total number of span-divisions for the flat slab in each of the Column strip and Field strip. The maximum number of elements per row is 5 based on the 5 distribution factors used. | | | | | | | | | | | | | | | | | | | | | | | | | | | | | | |  |  |  |  |
|  | | | | | | | $A_{smin}= \left( \frac{0.6}{F_{y}} \right)*B*d$ | | | | | | | | | | | | | | | | [26] | | | | | | | | | | | |
| where B is the effective horizontal distance which is equal 1000 mm in case of slabs. | | | | | | | | | | | | | | | | | | | | | | | | | | | | | | | | | | |
|  | | | | | | | $A_{s}CS= \left[ \begin{matrix} {AC}_{11} & {AC}_{12} & \cdots& {AC}_{15} \\ {AC}_{21} & {AC}_{22} & \cdots& {AC}_{25} \\ \vdots& \vdots& \ddots& \vdots\\ {AC}_{n1} & {AC}_{n2} & \cdots& {AC}_{n5} \end{matrix} \right]$ | | | | | | | | | | | | | | | | [27] | | | | | | | | | | | |
|  | | | | | | | $A_{s}FS= \left[ \begin{matrix} {AF}_{11} & {AF}_{12} & \cdots& {AF}_{15} \\ {AF}_{21} & {AF}_{22} & \cdots& {AF}_{25} \\ \vdots& \vdots& \ddots& \vdots\\ {AF}_{n1} & {AF}_{n2} & \cdots& {AF}_{n5} \end{matrix} \right]$ | | | | | | | | | | | | | | | | [28] | | | | | | | | | | | |
| where n is the total number of span-divisions for the flat slab in each of the column strip and field strip. The maximum number of elements per row is 5 based on the 5 distribution factors used. | | | | | | | | | | | | | | | | | | | | | | | | | | | | | | | | | | |
| Type of Column | | | | | | | | | | | | | | Interior | Exterior | | | | Corner | | | | | |  | | | | | |  |  |  |  |
| a_1_ | | | | | | | | | | | | | | $a+d$ | $a+d$ | | | | $a+\left( \frac{d}{2} \right)$ | | | | | | [29] | | | | | |  |  |  |  |
| b_1_ | | | | | | | | | | | | | | $b+d$ | $b+\left( \frac{d}{2} \right)$ | | | | $b+\left( \frac{d}{2} \right)$ | | | | | | [30] | | | | | |  |  |  |  |
| Perimeter | | | | | | | | | | | | | | $2 (a_{1}+b_{1})$ | $a_{1}+{2b}_{1}$ | | | | $a_{1}+b_{1}$ | | | | | | [31] | | | | | |  |  |  |  |
| Q_UP_ | | | | | | | | | | | | | | $W_{u} \left( \left( L_{1}L_{2} \right)-\left( a_{1}b_{1} \right) \right)$ | $W_{u} \left( \left( \frac{L_{1}L_{2}}{2} \right)-\left( a_{1}b_{1} \right) \right)$ | | | | $W_{u} \left( \left( \frac{L_{1}L_{2}}{4} \right)-\left( a_{1}b_{1} \right) \right)$ | | | | | | [32] | | | | | |  |  |  |  |
| where *d is the effective slab depth and a_1_ and b_1_ are the critical section dimensions.* | | | | | | | | | | | | | | | | | | | | | | | | | | | | | | |  |  |  |  |
|  | | | | | | | $q_{up}= \frac{\left( Q_{UP}*\beta\right)}{\left( b_{O}*d \right)}$ | | | | | | | | | | | | | | | | | | [33] | | | | | |  |  |  |  |
| where *β is a factor accounting for additional punching shear stresses due to moment transfer from slabs to columns with values (1.15, 1.30 and 1.50) depending on the column type and b_O_ is the critical shear perimeter.* | | | | | | | | | | | | | | | | | | | | | | | | | | | | | | |  |  |  |  |
|  | | $q_{CUP}=0.316 \left( 0.5+\frac{a}{b} \right)*\sqrt{\frac{f_{cu}}{\lambda_{c}}}$ | | | | | | | | | | | | | | | | | | [34] | | | | | | | | | | |  |  |  |  |
|  | | $q_{CUP}=0.8 (\frac{\alpha*d}{b_{O}}+0.2)*\sqrt{\frac{f_{cu}}{\lambda_{c}}}$ | | | | | | | | | | | | | | | | | | [35] | | | | | | | | | | |  |  |  |  |
|  | | $q_{CUP}=\begin{aligned} 0.316 \sqrt{\frac{f_{cu}}{\lambda_{c}}}, &\leq1.6 N/{{mm}^{2}} \end{aligned}$ | | | | | | | | | | | | | | | | | | [36] | | | | | | | | | | |  |  |  |  |
|  | | | | | | | | | | ${BeamLoads}_{X}= \left[ \begin{matrix} {PC}_{11} & {PC}_{1m} \\ {PC}_{21} & {PC}_{2m} \\ \vdots& \vdots\\ {PC}_{n1} & {PC}_{nm} \end{matrix} \right]$ | | | | | | | | | | | | | | | | [37] | | | | |  |  |  |  |
|  | | | | | | | | | | ${BeamLoads}_{Y}= \left[ \begin{matrix} {PC}_{11} & {PC}_{1m} \\ {PC}_{21} & {PC}_{2m} \\ \vdots& \vdots\\ {PC}_{n1} & {PC}_{nm} \end{matrix} \right]$ | | | | | | | | | | | | | | | | [38] | | | | |  |  |  |  |
| where n is the total number of beams in the plan and m is the number of loads carried by each beam (m can be 1 or 2; 2 in case of internal beams and 1 in case of external beams). | | | | | | | | | | | | | | | | | | | | | | | | | | | | | | |  |  |  |  |
|  | | | | | | | | | $I=\frac{b_{1}* \left( \frac{L_{1}}{12} \right)^{3}}{12}$ | | | | | | | | | | | | | | | | [39] | | | |  |  |  |  |  |  |
| where b_1_ is the width of the beam taken similar to wall thickness and L_1_ is the span of the beam. The depth of the beam is initially assumed 1/12 of the beam span. | | | | | | | | | | | | | | | | | | | | | | | | | | | | |  |  |  |  |  |  |
|  | | | | | | | | | $I= \left[ \begin{matrix} {MI}_{11} & {MI}_{12} & \cdots& {MI}_{1m} \end{matrix} \right]$ | | | | | | | | | | | | | | | [40] | | | | |  |  |  |  |  |  |
| where m is the total number of axis in the X direction. | | | | | | | | | | | | | | | | | | | | | | | | | | | | |  |  |  |  |  |  |
|  | | | | | | | | | $K= \frac{I_{i}}{L_{j}}$ | | | | | | | | | | | | | | | [41] | | | | |  |  |  |  |  |  |
|  | | | | | | | | | $D.F= \frac{K_{i}}{\sum K_{s}}$ | | | | | | | | | | | | | | [42] | | | | | |  |  |  |  |  |  |
| where *K*_i_ is the stiffness factor of the member, K_S_ is the sum of stiffness factors of the members intersecting at the studied support. | | | | | | | | | | | | | | | | | | | | | | | | | | | | |  |  |  |  |  |  |
|  | | | | | | | | | $D.M= - \sum F.E.M, B.M, C.O.M$ | | | | | | | | | | | | | | [43] | | | | | |  |  |  |  |  |  |
| where *F.E.M is the Fixed End Moments, B.M. is the Balancing Moments and C.O.M is the carry over moment.* | | | | | | | | | | | | | | | | | | | | | | | | | | | | |  |  |  |  |  |  |
|  | | | | | | | | | | | $Final Support Moment=F.E.M+\sum D.M+ \sum C.O.M$ | | | | | | | | | | | | | | | | [44] | | | | |  |  |  |
| where D.M is the Unbalanced Distribution Moment and C.O.M is the carry over moment. | | | | | | | | | | | | | | | | | | | | | | | | | | | | | | | |  |  |  |
|  | | | | | | | | | | | $Final Midspan Moment={MS}_{1}- \left( \left( \frac{{MS}_{1}-{MS}_{2}}{2} \right)+\left( \frac{W*L^{2}}{8} \right) \right)$ | | | | | | | | | | | | | | | | [45] | | | | |  |  |  |
| where MS_1_ is the maximum moment at support per beam, MS2 is the minimum moment at support per beam, W is the total loads on beam and L is the length of the supported beam. | | | | | | | | | | | | | | | | | | | | | | | | | | | | | | | |  |  |  |
|  | | | | | | | | | $C= \frac{d}{\sqrt{\frac{M_{u}}{f_{cu}*b}}}$ | | | | | | | $A_{S}= \frac{M_{u}}{\left( f_{y}*J*d \right)}$ | | | | | [46], [47] | | | | | | | | | | |  |  |  |
| where *d is the effective depth of the beam (thickness- Cover specified by the code), f_cu_ is the concrete ultimate stress, b is the effective width of the compression zone of the slab and f_y_ is the steel yielding stress.* | | | | | | | | | | | | | | | | | | | | | | | | | | | | | | | |  |  |  |
|  | | | | | | | | $Total Loads on Beam=O.w of beam+Slab Load+Wall Loads$ | | | | | | | | | | | | | | | | [48] | | | |  |  |  |  |  |  |  |
| where *O.W is the own weight of the beam equals to (Beam cross section area * Unit load of concrete defined by the user).* | | | | | | | | | | | | | | | | | | | | | | | | | | | |  |  |  |  |  |  |  |
|  | | | | | | | | $DIR. V= \frac{W*L}{2}$ | | | | | | | | | | | | | | | | [49] | | | |  |  |  |  |  |  |  |
| where *W* is the total loads on beam and L is the Beam Length. | | | | | | | | | | | | | | | | | | | | | | | | | | | |  |  |  |  |  |  |  |
|  | | | | | | | | $AUX. V= \frac{\sum{MF}_{i}}{L_{i}}$ | | | | | | | | | | | | | | | | [50] | | | |  |  |  |  |  |  |  |
| where *M*F_i_ is the sum of the two bending moments at the right and left supports of each beam and L is the studied beam length. | | | | | | | | | | | | | | | | | | | | | | | | | | | |  |  |  |  |  |  |  |
|  | | | | | | | | $V.Reaction= \left( \sum DIR.V, AUX.V \right)_{Left}+\left( \sum DIR.V, AUX.V \right)_{Right}$ | | | | | | | | | | | | | | | | | [51] | | |  |  |  |  |  |  |  |
|  | | | | | | | | | $V.Reactions= \left[ \begin{matrix} {VR}_{11} & {VR}_{12} & \cdots& {VR}_{1m} \\ {VR}_{21} & {VR}_{22} & \cdots& {VR}_{2m} \\ \vdots& \vdots& \ddots& \vdots\\ {VR}_{n1} & {VR}_{n2} & \cdots& {VR}_{nm} \end{matrix} \right]$ | | | | | | | | | | | | | | | | [52] | | |  |  |  |  |  |  |  |
| where *n is the total number of axes in the studied direction and m is the number of supports per each axis*. | | | | | | | | | | | | | | | | | | | | | | | | | | | |  |  |  |  |  |  |  |

|  | | | | $q_{cu}= 0.16\sqrt{\frac{f_{cu}}{\lambda_{c}}}$ | $q_{u max}= 0.7\sqrt{\frac{f_{cu}}{\lambda_{c}}}$ | | [53], [54] | | | |
| --- | --- | --- | --- | --- | --- | --- | --- | --- | --- | --- |
| where $q_{cu}$*.is the shear stress for the uncracked concrete section,* $q_{u max}$ *is the maximum shear stress allowed for the reinforced concrete beams* and $\lambda_{c}$ *is factor of safety = 1.5.* | | | | | | | | | | |
|  | | | $q_{u}= \frac{V.Reactions}{b*d}$ | | | | | [55] | |  |
| *where b is the beam width and d is the clear depth of the beams.* | | | | | | | | | |  |
|  | | | | ${AS}_{s}=\left\{ \begin{aligned} Increase b {and}/{or d}, &q_{u}>q_{u max} \\ \frac{\left( q_{u}-0.12\sqrt{\frac{F_{cu}}{\lambda_{c}}} \right)*b*s*\lambda_{S}}{f_{y}},{q_{u max}>q}_{u}>q_{cu} \\ \frac{0.4*b*s}{f_{y}}, &q_{u}<q_{cu} \end{aligned} \right.$ | | | | [56] | |  |
| where *b is the beam width, d is the clear depth of the beam, s* *is the spacing between stirrups,* $\lambda_{c}$ *is factor of safety = 1.5, F_CU_ i*s the concrete ultimate compressive strength, $\lambda_{S}$ *is steel factor of safety =1.15 and F_y_ is the steel yielding stress.* | | | | | | | | | |  |
|  | | | $Critical Section Reduction Distance= \frac{d}{2}+ \frac{c}{2}$ | | | | | [57] | |  |
|  | | | | ${AS}_{s}= \left[ \begin{matrix} {AS}_{11} & {AS}_{12} & \cdots& {AS}_{1m} \\ {AS}_{21} & {AS}_{22} & \cdots& {AS}_{2m} \\ \vdots& \vdots& \ddots& \vdots\\ {AS}_{n1} & {AS}_{n2} & \cdots& {AS}_{nm} \end{matrix} \right]$ | | | | [58] | |  |
| where *n is the total number of axes in the studied direction and m is the number of supports per each axis*. | | | | | | | | | |  |
|  | | | ${AS}_{Ton}= {AS}_{m}*7.850$ | | | | | | [59] | |
|  | | ${{Variables}_{i} Limits}_{j}= \left[ \begin{matrix} {LI}_{11} & {LI}_{12} & \cdots& {LI}_{1m} \end{matrix} \right]_{ij}$ | | | | | [60] | | | |
| *where m is the number of dimensions in the studied X/ Y Directions and i is (Upper or Lower) and j is (X or Y Direction).* | | | | | | | | | | |
|  | | | ${Dimensions Random Variables}_{i}= \left[ \begin{matrix} {RN}_{11} & {RN}_{12} & \cdots& {RN}_{1m} \\ {RN}_{21} & {RN}_{22} & \cdots& {RN}_{2m} \\ \vdots& \vdots& \ddots& \vdots\\ {RN}_{n1} & {RN}_{n2} & \cdots& {RN}_{nm} \end{matrix} \right]$ | | | | | | [61] | |
| where *i is the studied X or Y direction, n is the total number of dimensions in the studied direction and m is the required size of population.* | | | | | | | | | | |
|  | | | ${Remapped RN}_{nm}= \frac{{RN}_{nm}}{\sum{RN}_{nm}per population per {Direction}_{i}}*L_{i}$ | | | | | | | [62] |
|  | | | $Optimization Random Variables= \left[ \begin{matrix} {RV}_{11} & {RV}_{12} & \cdots& {RV}_{1m} \\ {RV}_{21} & {RV}_{22} & \cdots& {RV}_{2m} \\ \vdots& \vdots& \ddots& \vdots\\ {RV}_{n1} & {RV}_{n2} & \cdots& {RV}_{nm} \\ 0 & 0 & \cdots& 0_{(n+1)m} \end{matrix} \right]$ | | | | | | | [63] |
| Where *n is the total number of dimensions in both X and Y directions where each RV is between the Upper and Lower Limits specified and m is the required size of population.* | | | | | | | | | | |
|  | | | $Optimization Results= \left[ \begin{matrix} {FD}_{11} & {FD}_{12} & \cdots& {FD}_{1m} \\ {FD}_{21} & {FD}_{22} & \cdots& {FD}_{2m} \\ \vdots& \vdots& \ddots& \vdots\\ {FD}_{n1} & {FD}_{n2} & \cdots& {FD}_{nm} \\ {OR}_{\left( n+1 \right)1} & {OR}_{\left( n+1 \right)2} & \cdots& {0R}_{\left( n+1 \right)m} \\ {OR}_{\left( n+2 \right)1} & {OR}_{\left( n+2 \right)2} & \cdots& {OR}_{\left( n+2 \right)m} \\ \vdots& \vdots& \ddots& \vdots\\ {OR}_{\left( n+i \right)1} & {OR}_{\left( n+i \right)2} & \cdots& {OR}_{\left( n+i \right)m} \end{matrix} \right]$ | | | | | | | [64] |
| Where *FD is final optimum dimensions, OR is the Final corresponding optimum objective Function results, n is the total number of dimensions in both X and Y directions, m is the required size of population and i is the number of objectives required.* | | | | | | | | | | |
